# Composition and geographic variation of the bacterial microbiota associated with the coelomic fluid of the sea urchin *Paracentrotus lividus*

**DOI:** 10.1038/s41598-020-78534-5

**Published:** 2020-12-08

**Authors:** Teresa Faddetta, Francesco Ardizzone, Francesca Faillaci, Chiara Reina, Emilia Palazzotto, Francesco Strati, Carlotta De Filippo, Giovanni Spinelli, Anna Maria Puglia, Giuseppe Gallo, Vincenzo Cavalieri

**Affiliations:** 1 Department of Biological, Chemical and Pharmaceutical Sciences and Technologies, University of Palermo, viale delle Scienze, ed. 16, 90128 Palermo, Italy; 2 Department of Health Promotion, Mother and Child Care, Internal Medicine and Medical Specialties (PROMISE), University of Palermo, Piazza Delle Cliniche 2, 90127 Palermo, Italy; 3 Department of Experimental Oncology, European Institute of Oncology, Via Adamello, 16, 20139 Milano, Italy; 4 Institute of Agricultural Biology and Biotechnology, National Research Council, Via Moruzzi, 1, 56124 Pisa, Italy

**Keywords:** Microbiology, Microbial communities

## Abstract

In the present work, culture-based and culture-independent investigations were performed to determine the microbiota structure of the coelomic fluid of Mediterranean sea urchin *Paracentrotus lividus* individuals collected from two distinct geographical sites neighboring a high-density population bay and a nature reserve, respectively. Next Generation Sequencing analysis of 16S rRNA gene (rDNA) showed that members of the *Proteobacteria*, *Bacteroidetes* and *Fusobacteria* phyla, which have been previously reported to be commonly retrieved from marine invertebrates, dominate the overall population of microorganisms colonizing this liquid tissue, with minority bacterial genera exhibiting remarkable differences among individuals. Our results showed that there is a correlation between microbiota structure and geographical location of the echinoderm collection site, highlighting over-representation of metagenomic functions related to amino acid and bioactive peptides metabolism in specimens inhabiting the nature reserve. Finally, we also described the developmental delay and aberrations exhibited by sea urchin embryos exposed to distinct bacterial isolates, and showed that these defects rely upon hydrophilic compound(s) synthesized by the bacterial strains assayed. Altogether, our findings lay the groundwork to decipher the relationships of bacteria with sea urchins in their aquatic environment, also providing an additional layer of information to understand the biological roles of the coelomic fluid.

## Introduction

Life on earth began in the oceans. Over millions of years, the organisms that inhabit the seas evolved strategies to handle a changing and sometimes invasive environment. Extending through a dynamic interaction from antagonistic to cooperative elements, marine eukaryotic organisms became hosts for large and complex community of microorganisms playing important roles in nutrition^1^, response to environmental stimuli^2,3^, disease-resistance and evolution^4^, as well as chemical defence against both predators and competitors^5,6^. In fact, the secondary metabolites produced by the symbionts include compounds showing anti-inflammatory, antitumor, antibiotic and antifungal activities, which are particularly interesting in drug discovery campaigns (reviewed in^7^).

Studies of bacterial communities associated with body tissues of marine animals have largely focused on sponges, corals, bryozoans and crustaceans^8–12^. Despite the scientific and economic importance of sea urchins, little attention has been given to their microbial community and the potential role of microorganisms in the host physiology. In particular, some reports focused on the microbial abundance and diversity of the digestive system of distinct species, such as *Echinocardium cordatum*^13^, *Paracentrotus lividus*^14^, and *Lytechinus variegatus*^15,16^, while few other works focused on the general role of microorganisms in nutrients digestion^13,17^ or in disease progression^18,19^.

Sea urchins are invertebrate animals distributed throughout the oceans of the world, where they represent central players in many benthic ecosystems. Besides their ecological role and economic importance as fishery products^20^, sea urchins have been used as model organisms for biological research for nearly two centuries, contributing significantly to our understanding of fertilization, molecular embryology, gene regulation, cell cycle regulation, evolution, population genetics, and toxicology^21–26^. The reason of their extensive use as experimental model likely relies in their phylogenetic position. Sea urchins are indeed basal deuterostomes belonging to Echinodermata, the sister phylum to the chordates^27^, and therefore they are closely related to humans^28^.

Similar to the blood of higher metazoans, the main circulatory medium of sea urchins is the coelomic fluid enclosed in the main body cavity. Such an internal fluid contains a heterogeneous mixture of organic molecules and circulating cells that ensures essential functions such as nutrient transport and immune activity^29–31^. The coelomic fluid is in equilibrium with the surrounding seawater environment and in direct contact with internal tissues, whereas the circulating immunocytes respond to pathogen challenge producing antimicrobial molecules^32–34^. Therefore, the coelomic fluid could be considered a rather complex tissue that mediates responses to wounding and microbial infections by undergoing reactions such as opsonization, coagulation, encapsulation and phagocytosis^34^. In light of this, the coelomic fluid of these echinoderms has been traditionally supposed to be a microorganism-free compartment^35,36^. Contrarily, recent reports described the identification of microbial communities associated with the coelomic fluid of holothurians, starfish and sea urchins^37–43^, suggesting that complex symbiotic associations could exist in such a perivisceral liquid of the echinoderms.

In the present study, we confirm this hypothesis describing the composition of the bacterial microbiota in the coelomic fluid from the Mediterranean sea urchin *Paracentrotus lividus*.

## Results

### Structure of the coelomic fluid bacterial microbiota of *P. lividus*

In order to collect sea urchin *P. lividus* specimens, two distinct geographical sites, arbitrarily named A and B, were chosen for their proximity to a high-density population bay and to a nature reserve, respectively. Based on the notion that the cultivability of marine microbial communities often ranges below 10% of total bacteria^44,45^, to obtain a comprehensive understanding of the microbiota structure we performed an analysis based on Next Generation Sequencing of V3-V4 region amplicons of 16S rDNA obtained from metagenomic DNA extracted from coelomic fluid of six *P. lividus* individuals from site A and four from site B (Supplementary Fig. S1, Table S1). Overall, this approach led to the identification of 227 genera of which 38 and 68 were found exclusively in samples derived from sites A and B, respectively (Supplementary Table S1, Fig. S2). The analysis of alpha-diversity (*i.e*. the ecological richness within each sample) revealed a variation (*p* < 0.05) in the number of observed Operational Taxonomic Units (OTUs) and Shannon entropy index (*p* < 0.01) among the samples collected in A and B sites, with B showing a diversity greater than A (Supplementary Fig. S3). The analysis of beta-diversity highlighted that the microbial community structure within the coelomic fluid of echinoderms collected from site A differs significantly with respect to that of individuals from site B (Supplementary Fig. S1), as revealed by weighed Unifrac and Bray–Curtis distances (*p* = 0.057 and *p* = 0.025, respectively).

At the phylum level, a predominance of taxa belonging to *Proteobacteria*, *Bacteroidetes* and *Fusobacteria* was observed in the microbiota of all specimens collected from either A or B sites, accounting for about 90% of abundance per each individual (Supplementary Fig. S1; Table S1). On the contrary, minority phyla (i.e. having a relative abundance lower than 1%) were overtly different among individuals (Supplementary Fig. S1).

### Geographical variation of coelomic fluid bacterial microbiota composition

**Figure 1:**
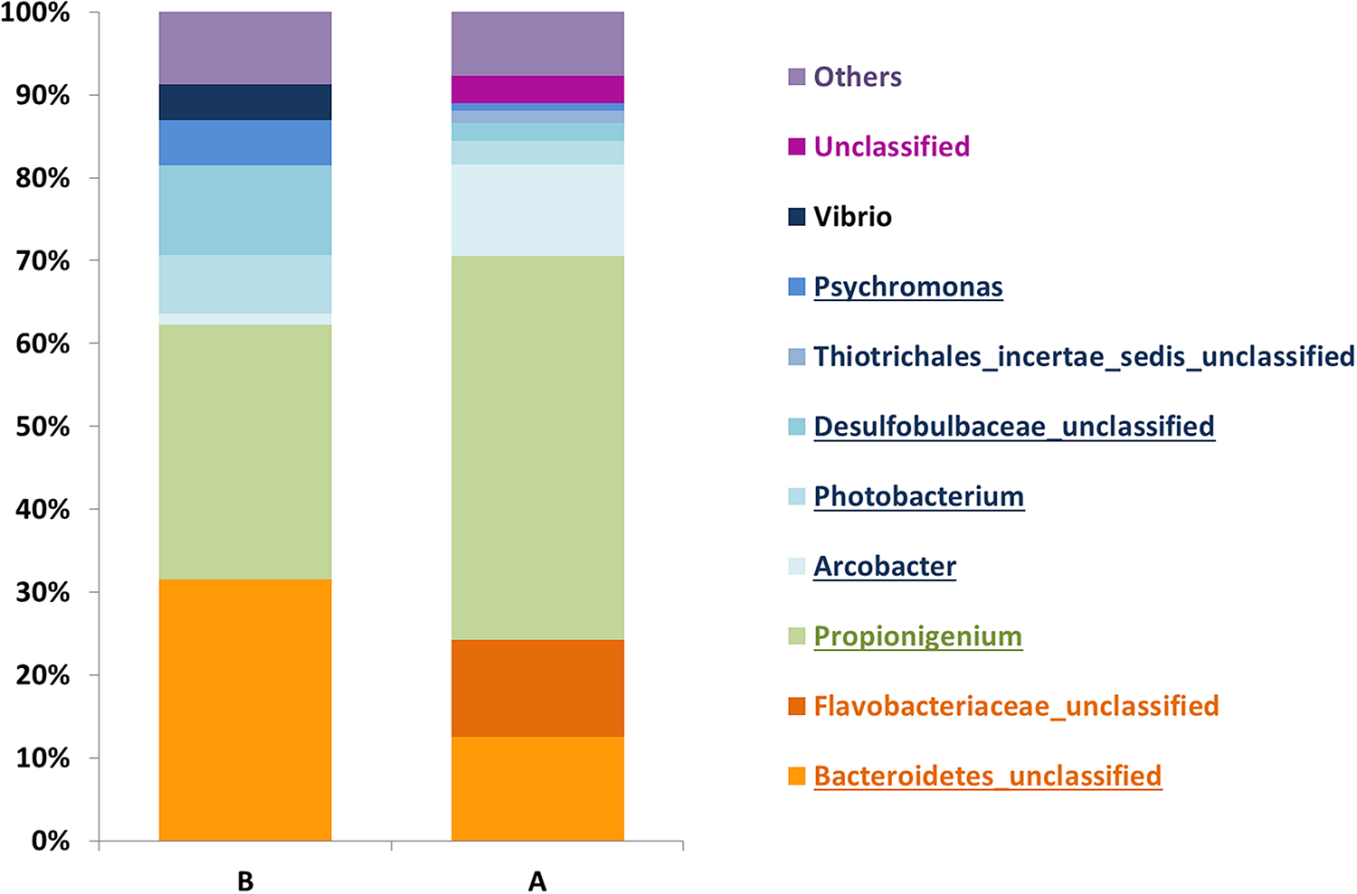
Mean relative abundances of taxa, at genus level, of the coelomic fluid microbiota of *P. lividus* individuals collected from sites A and B. The corresponding phylum is highlighted by using shades of blue, green, and orange for *Proteobacteria*, *Fusobacteria* and *Bacteroidetes*, respectively. All bacterial genera with relative abundance < 1% are reported together and labeled as “Others”. The underlined taxa represent the microbiota core members.

Looking at the average relative abundance, the most abundant genera (*i.e.* > 1.0%) in B site samples belonged to unclassified genera of *Bacteroidetes* phylum (31.6%) and of *Desulfobulbaceae* family (10.1%), and to *Propionigenium* (30.7%), *Photobacterium* (7.1%), *Psychromonas* (5,4%), *Vibrio* (4.3%) and *Arcobacter* (1.3%) genera. Using the same criterion, the most abundant bacteria in A site samples belonged to an unclassified phylum (3.3%) and to unclassified genera of *Bacteroidetes* phylum (12.5%), of *Flavobacteriaceae* (11.7%), of *Desulfobulbaceae* (2.2%) and of *Thiotrichales incertae sedis* families (1.4%), and to *Propionigenium* (46.3%), *Arcobacter* (11.1%), *Photobacterium* and *Psychromonas* (1.0%) (Fig. 1). It follows that a core in bacterial microbiota structure can be defined by the sum of the majority taxa (*i.e*. having an average relative abundance higher than 1%), as they collectively account for more than 75% in the relative abundance, irrespective of specimen collection site. In particular, such a core comprises the unclassified genera of *Bacteroidetes* phylum and *Desulfobulbaceae* family, and the *Propionigenium*, *Photobacterium*, *Psychromonas* and *Arcobacter* genera (Fig. 1).

**Figure 2:**
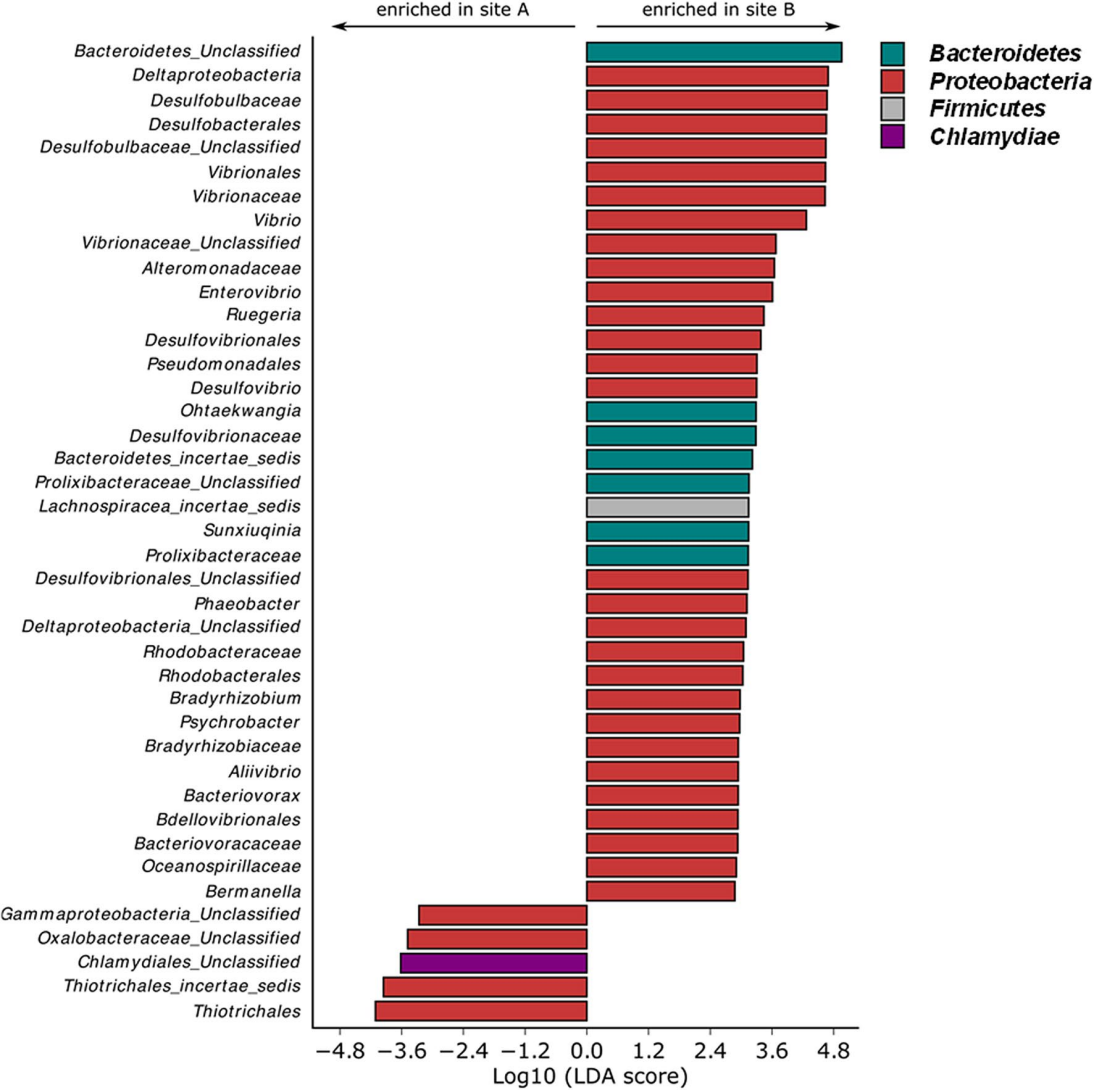
Comparative analysis of the most discriminant bacterial taxa in the coelomic microbiota of *P. lividus* individuals from site A vs site B. Log_10_ of LDA scores was calculated for the most discriminant bacterial taxa identified by LEfSe. Positive and negative LDA scores indicate the taxa enriched in the coelomic microbiota of *P. lividus* specimens from A and site B sites, respectively. Only taxa having a *p* < 0.05 (Wilcoxon rank-sum test) and LDA >|2.0| are shown.

The LEfSe analysis, revealing the majority bacterial genera that characterize A and B sites based on their relative abundance, highlighted 36 and 5 taxa in A and B sites, respectively (Fig. 2). Interestingly, besides the unclassified genera of *Bacteroidetes* and *Desulfobulbaceae* family (in samples from site B), and the unclassified genus of *Thiotrichales incertae sedis* family (in samples from site A), specific minority genera (i.e. having relative abundance lower than 1.0%) significantly enriched in A and B sites mostly belonged to the *Proteobacteria* phylum.

### Metabolic functions associated to the coelomic fluid microbiota

**Figure 3:**
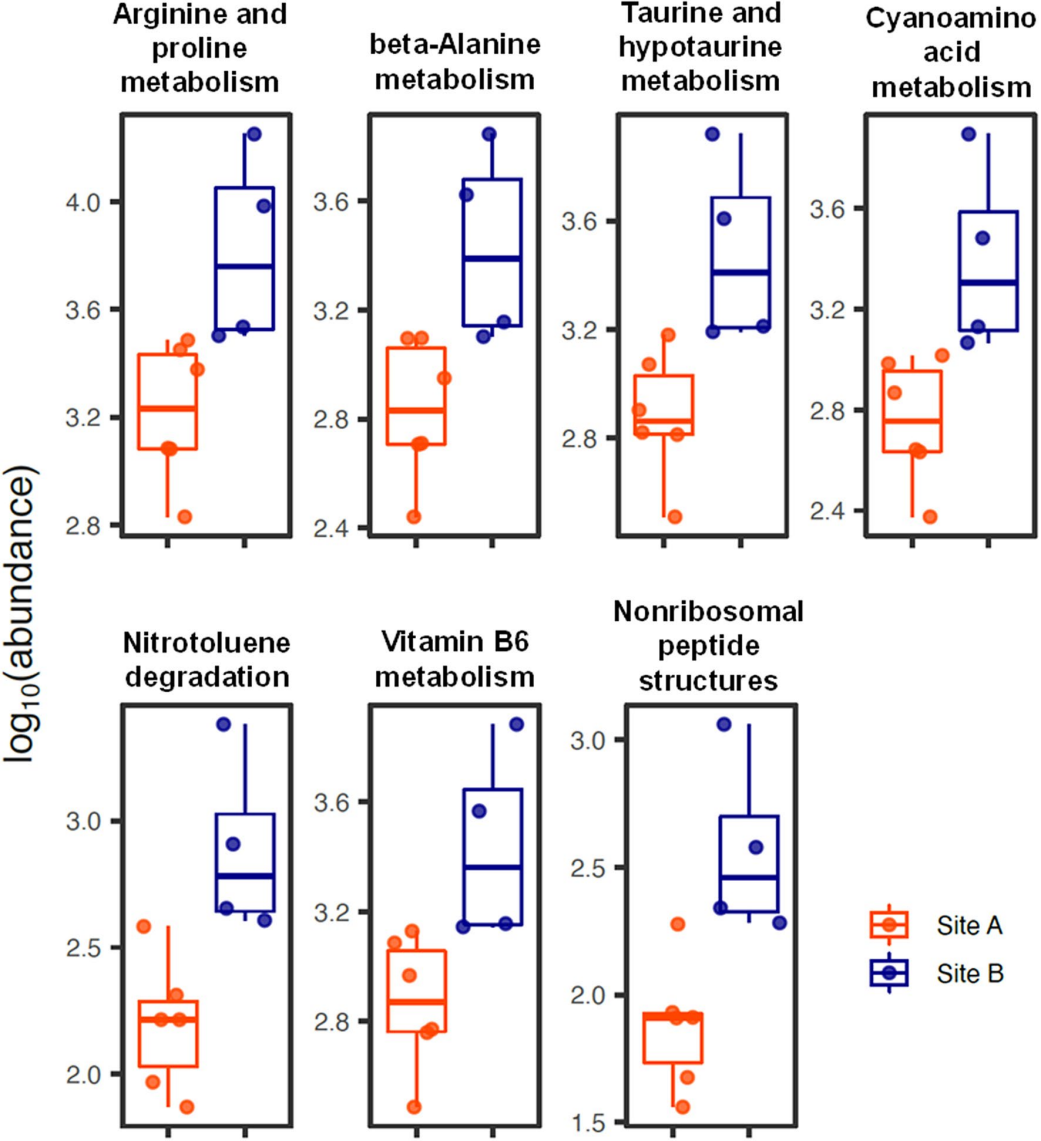
Putative functional capabilities differentially represented in the coelomic microbiota of specimens from A and B sites. A total of 7 differentially represented (*p* < 0.01) metabolic pathways were inferred by Piphillin using the KEGG database^80^.

The Piphillin tool^46^ was used to examine the putative functional capabilities of the coelomic fluid microbiota of the *P. lividus* specimens collected from both the above-mentioned geographical sites, inferring the abundance of functional genes from 16S rDNA counts. Including only KEGG metabolic pathways of bacteria, 7 metagenomic functions were found to be differentially abundant (*p* < 0.01) between the microbiota of individuals collected from A and B sites. In particular, they were all over-represented in B site specimens, whereas 4 of them (arginine and proline metabolism, beta-alanine metabolism, taurine and hypotaurine metabolism, and cyanoamino acid metabolism) pertained the amino acid metabolism, and 1 (nonribosomal peptide structures) dealt with the synthesis of bioactive peptides (Fig. 3).

### Isolation and phylogenetic characterization of bacteria inhabiting the coelomic fluid of *P. lividus*

**Figure 4:**
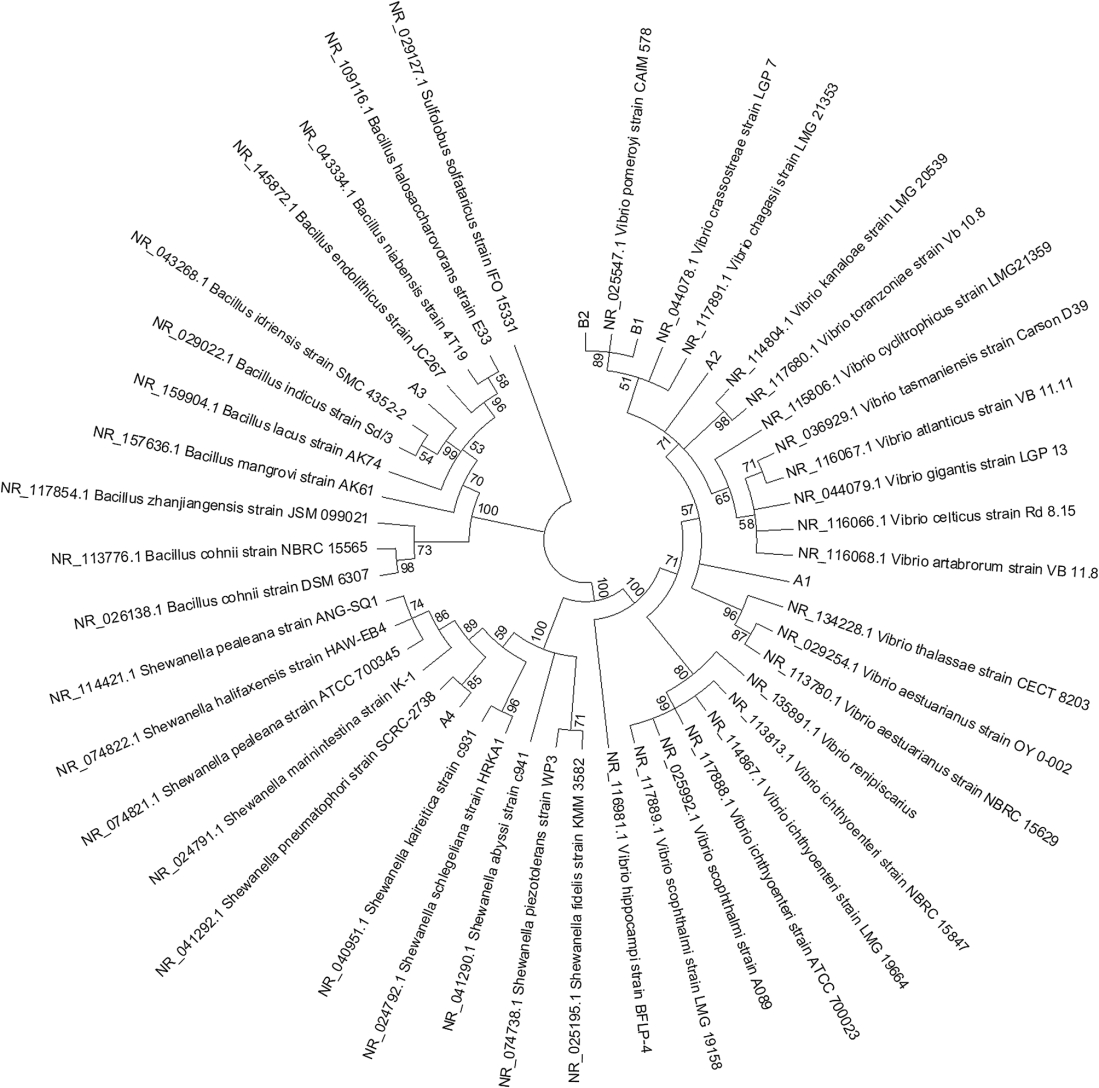
Optimal phylogenetic tree of the six cultivable bacterial strains isolated from coelomic fluid of *P. lividus* specimens collected in A (A1–4) and B (B1–2) sites. The analysis involved 48 nucleotide sequences. The evolutionary history and distances were obtained using neighbor-joining and the maximum composite likelihood methods, respectively. Distances are in the units of the number of base substitutions per site.

A culture-based investigation of the bacterial populations inhabiting the coelomic fluid of individuals collected from both the mentioned sites revealed an amount of 10^4^–10^5^ bacterial isolates/mL. Among these, a total of 70 colonies were picked and re-streaked on fresh marine agar plates. Bacterial isolates that shared similar colony morphology were grouped together and considered to be redundant. Then, a total of six isolates (namely A1-4 and B1-2 from A and B sites, respectively), each exhibiting representative colony morphology and/or pigmentation, were selected for phylogenetic analysis based on 16S rDNA sequence (Fig. 4). This analysis confirmed that the six isolates represented bacterial strains commonly found in marine organisms, with four isolates (A1, A2, B1 and B2) belonging to *Vibrio* genus and two isolates (A3 and A4) belonging to *Bacillus* and *Shewanella* genera, respectively.

### Developmental effects of coelomic fluid bacterial isolates on *P. lividus*

**Figure 5:**
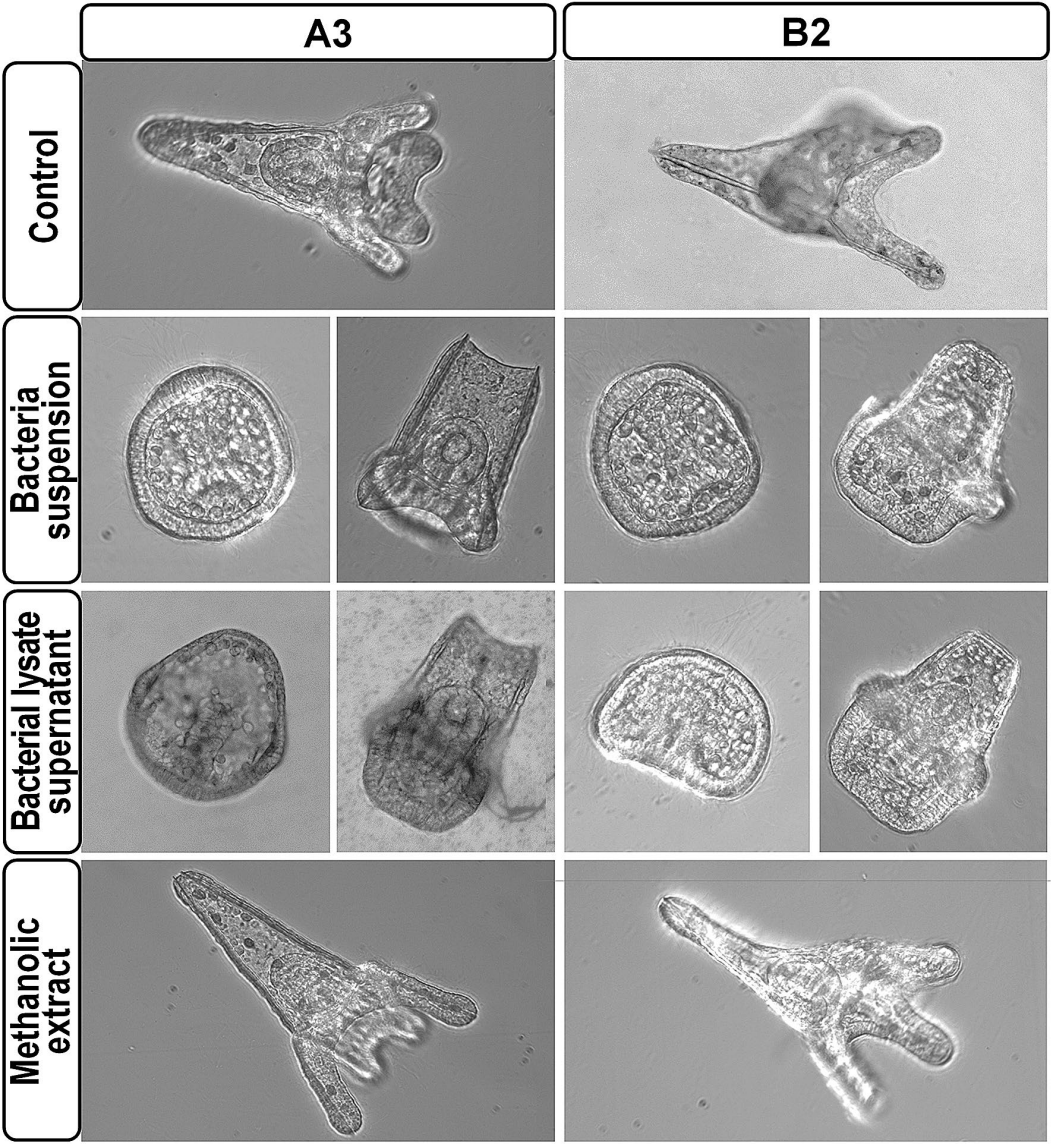
Developmental effects of coelomic fluid bacterial strain exposure on *P. lividus*. Representative embryos cultured in the absence (control) or in the presence of the indicated bacterial strains, bacterial lysate supernatants or equivalent amounts of methanolic extracts, and observed at the pluteus stage.

Since the bacterial strains isolated by culture-dependent analysis belong to genera whose members are capable to influence distinct health and physiological mechanisms of marine organisms^43,47–49^, we attempted to explore their effects on embryogenesis of *P. lividus*. Thus, sea urchin zygotes were cultured continuously after fertilization in the presence of intact bacteria resuspended in Millipore-filtered sea water (MFSW) at concentrations of 1 × 10^6^/mL and 1 × 10^7^/mL, or with equivalent amounts of bacterial lysate supernatants or methanolic extracts. In this analysis, we focused on *Bacillus* (A3) and *Vibrio* (B2) isolates that were randomly selected as representative of the two geographic sites described. Treated sea urchin embryos were observed at several time intervals during development, their overall morphology was examined and compared to that of control unperturbed embryos from the same batches. Development of treated embryos was apparently normal during early cleavage and until the mesenchyme blastula stage. From this stage onwards, significant fractions of the embryos treated with the bacteria suspensions showed overt developmental delay and defects (70%), as well as arrested development (20%). In particular, the morphology of most of these embryos remained roughly spherical during development, never evolving into the characteristic easel-like shape exhibited by control plutei (Fig. 5). Nonetheless, most of the assayed specimens eventually produced mesenchyme cells, archenteron and skeletal elements similar to control larvae (Fig. 5), indicating that both morphogenetic movements and specification of main tissues were not impaired by the exposure to the two bacterial strains examined. Intriguingly, in a small but reproducible fraction (~ 15%) of these embryos the body rod spicules extended parallel instead of converging toward the aboral apex, giving the embryos the characteristic shape of a chair (Fig. 5). All the observed anomalies arose in a dose-dependent manner, irrespectively of the bacterial strain. Worth to mention, exactly the same phenotypes were obtained following exposure of embryos with amounts of bacterial lysate supernatants corresponding to 1 × 10^7^ cells/mL (Fig. 5), suggesting that the observed aberrations on embryogenesis rely upon hydrophilic compound(s) synthesized by the bacterial strains assayed. In support of this hypothesis, sibling embryos exposed to equivalent amounts of either methanolic extracts (Fig. 5) or methanolic hydrochloric acid, as well as PBS, developed normally compared to control unperturbed embryos. We also noticed that the swimming behaviour of larvae treated with either bacterial strains or the corresponding bacterial lysate supernatants changed markedly, ranging from abnormal locomotion to immobilization. In particular, while all of the control larvae did swim freely exhibiting rapid forward movements throughout the water column, roughly 50% and 80% of the embryos exposed to *Bacillus* (A3) and *Vibrio* (B2), respectively, were turning around their axis or circling slowly at the bottom of the culture plate.

## Discussion

Individual asymmetries in the composition of the bacterial microbiota associated to distinct metazoans, spanning from *Hydra* to mammalians, have been reported by several authors^50–52^. For instance, Enomoto et al.^37^ reported a distinctive bacterial microbiota structure in the coelomic fluid of distinct holothurian individuals. These findings, coupled to the fact that the perivisceral liquid of echinoderms is in dynamic equilibrium with the surrounding seawater environment^53^, raise questions about the stability of the microbiota composition in the coelomic fluid of individual sea urchins across seasons and geographic sites. This point has been addressed in the present study by describing and comparatively analysing the bacterial microbiota residing in the coelomic fluid of sea urchins collected from two distinct geographical sites. Indeed, this analysis revealed that a core bacterial microbiota formed by members of the *Proteobacteria*, *Bacterioidetes* and *Fusobacteria* phyla is established in all the analyzed specimens, irrespective of the sampling site. On the other hand, minority taxa characteristically related to the geographical localization constituted the most variable part of the coelomic fluid bacterial microbiota structure. These findings could reflect environmental and physiological stimuli to each *P. lividus* individual, which can be highly variable in close subcostal areas of heavily anthropized regions. In strict accordance, the examination of differentially abundant metabolic pathways, inferred from 16S rDNA counts by a Piphillin-based analysis using the KEGG metabolic database, highlighted a possible over-representation of metabolic functions associated with amino acid metabolism and production of bioactive peptides in the *P. lividus* coelomic bacterial microbiota of site B specimens. Although this finding suggests that different metabolic functions are associated with the bacterial microbiota of specimens residing in site A and B, the exact role of these functional differences in the environmental contest is still elusive and, therefore, further dedicated studies are required to underline the crucial importance of bacteria-host association dynamics in the aquatic environment.

Worth to mention, all the taxa identified in the present study have been previously described as commonly associated with coastal marine ecosystems and especially with marine invertebrates, potentially having important metabolic and physiological roles in their host species^54,55^. For example, bacteria belonging to the *Vibrio* and *Photobacterium* genera are among the most recurring taxa in marine metazoan microbiotas, whereas their association may be (i) symbiotic^56^, (ii) neutral as commensal microorganisms^57^, or (iii) parasitic as agents of disease^58^. Bacteria belonging to *Propionigenium* genus were found associated with tyrian purple producing gland in the marine gastropod *Dicathais orbita*^59^, and the *P. maris* species is characterized for the capability of debrominating biogenic bromoaromatic compounds that are often distributed in intertidal marine sediments^60^. *Desulphovibrio*, usually found in marine sediments, was also revealed to be part of the sponge microbiota, having the characteristic property to conduct anaerobic reduction thus recycling marine inorganic elements^61^.

Irrespective of the enormous amount of microbial community data from the coelomic fluid, the mechanism of symbiotic establishment and its exact role within the sea urchin host remains mostly unclear. Establishment of symbiotic association in marine organisms has been recognized as a key feature for the correct differentiation of tissues and organs during embryogenesis^56,62–65^. The paradigmatic example is given by the functional maturation of the light organ of the Hawaiian bobtail squid *Euprymna scolopes*, which requires specific colonization by the luminous bacterium *Vibrio fischeri* during embryonic development^56,62^. Moreover, a pervasive example of microbial signalling in marine invertebrate development is the induction of metamorphosis and settlement of the benthonic larvae^63^. This transition is absolutely required for the completion of the life cycle of these animals, and in the case of sea urchins it roughly coincides with the beginning of coelomic fluid formation^29^. In light of this, an intriguing hypothesis is that specific bacterial colonization of the coelomic fluid could occur at the critical time of larval settlement. Thus, although a non-mutualistic partnership cannot be excluded, it could be reasonably supposed that the microbial–host interaction may provide sea urchins with benefits that include, among others, the interchange and assimilation of nutrients as well as defence barrier against pathogens. Another fascinating role in the host speciation and evolution processes could be potentially played by the bacteria of the *Bacteroidetes* phylum, which have been associated with alterations of the host reproduction^64,65^.

The six bacterial strains that we isolated by culture-dependent analysis belong to *Vibrio*, *Bacillus* and *Shewanella* genera, respectively, whose members are notoriously capable of: (i) influencing immune cell behaviour in marine invertebrates, as in the case of the *Vibrio* spp.^66^; (ii) producing biologically active metabolites and/or small molecules and enzymes, as in the case of *Bacillus*^67^; (iii) detoxifying environments due to their metabolic versatility, as in the case of *Shewanella*^68^. Therefore, these strains offer the unique possibility to be characterized for their possible roles in *P. lividus* development and physiology, as well as for potential use in biotechnological applications. Due to the importance of sea urchins in many scientific and economical fields like molecular embryology, marine ecology and fishery, detailed information on this issue is of outstanding interest.

In the present study, we show that a significant fraction of developing embryos exposed to either *Bacillus* (A3) or *Vibrio* (B2) bacterial strains isolated from coelomic fluid samples exhibited similar developmental delay and aberrations. The observed effects could depend on the bacterial doses used in our assay, which are presumably much higher than those encountered in the ocean by free-living echinoderm embryos. In support of this hypothesis, previous reports showed that many bacterial strains normally associate with healthy sea urchin larvae, where they behave as commensal or mutualistic microorganisms (reviewed in^69^). However, under dysbiosis conditions an excessive bacterial load can overwhelm larval defenses, thereby affecting larval welfare and physiology^66,70^. Moreover, though *Bacillus* (A3) and *Vibrio* (B2), as well as many other bacterial strains, inhabit the coelomic fluid of *P. lividus*, it should be emphasized that sea urchin larvae and juveniles in aquaculture facilities are apparently more susceptible to bacterial infections than adult echinoderms^71^.

In conclusion, our results not only provide an unexplored and unexploited source of microbial diversity, but also could be helpful for decoding the biological functions of the echinoderm coelomic fluid, for understanding the roles of bacteria in the physiology and ecology of echinoderms, and for the improvement of their aquaculture conditions.

## Methods

### Collection of sea urchins and DNA extraction from coelomic fluid

To counterbalance possible differences in the composition of the coelomic fluid due to different marine microenvironments, adult *P. lividus* specimens were collected during Spring 2015 from two distinct localities around the Eastern coast of Palermo (site A: 38.11° N 13.49° E, and site B: 38.21° N 13.24° E, respectively), chosen for their proximity to a high-density population urban area and to a nature reserve, respectively. Sampled individuals were transported in thermally insulated containers filled with cold sea water, and they arrived to the laboratory within one hour since collection. Immediately after, sampled individuals were rinsed thoroughly with sterile 0.45 µm-filtered sea water and kept in aseptic conditions. Six and four individuals (mean test diameter ranging between 55.0 ± 2.0 mm) from site A and B, respectively, were randomly selected and subjected to further analysis as follows. Approximately 7 mL of coelomic fluid were drawn, through the peristomial membrane surrounding the Aristotele’s lantern, using a sterile syringe with a 21-gauge needle, and 6.5 mM EDTA pH 8.0 was immediately added to prevent clot formation. The coelomic fluid was centrifuged at 6000 × *g* for 10 min at 4 °C in an Eppendorf 5804R centrifuge, and DNA extracted from the resulting cellular component by using the Genomic DNA purification kit (Thermo Scientific), following the manufacturer’s recommendations.

### Generation of 16S rDNA amplicon library, next generation sequencing and bioinformatic analysis

The quality of metagenomic DNA samples extracted from coelomic fluid was preliminarily checked by end point PCR to amplify genes encoding 16S rDNA. PCR reactions were performed using 100 ng of DNA as template in 1X PCR RxN Reaction Buffer (Invitrogen), 3.5 mM MgCl2, 200 µM dNTPs (Invitrogen), 0.2 µM universal primers 27F (5′-AGAGTTTGATCMTGGCTCAG-3′) and 1492R (5′-TACGGYTACCTTGTTACGACTT-3′) and 1 U of Taq DNA Polymerase Recombinant (Invitrogen). Thermal cycling conditions were 94 °C for 3 min, followed by 30 cycles of 94 °C for 45 s, 50 °C for 1 min and 72 °C for 90 s, and finally 72 °C for 10 min. According to PCR-based quality test, the metagenomic DNA of 10 samples (6 from A site and 4 from B site) was considered suitable for NGS analysis that was performed by IGA Technology Services (Udine, Italy) using MiSeq (Illumina, San Diego, CA, USA) to generate 300 bp paired-end reads of 16S rDNA V3-V4 region (FWD: 5′-CCTACGGGNGGCWGCAG-3′; REV: 5′-GACTACHVGGGTATCTAATCC-3′). NGS data are available at the European Nucleotide Archive (https://www.ebi.ac.uk/ena) with the accession identifier PRJEB27034.

Reads were pre-processed using the MICCA pipeline v 1.5.0^72^, and the overlapping paired-end reads were merged using micca mergepairs^73^. Forward and reverse primer trimming and quality filtering were performed using micca trim and micca filter, respectively. De novo greedy clustering and chimera filtering were performed by using micca otu: operational taxonomic units (OTUs) were assigned by clustering the sequences with a threshold of 97% pairwise identity, and their representative sequences were classified using micca classify with the RDP classifier v2.11^74^. Multiple sequence alignment was performed using the Nearest Alignment Space Termination (NAST)^75^ algorithm implemented in micca msa with the template alignment clustered at 97% similarity of the Greengenes database (release 13_05)^76^. The phylogenetic tree was inferred using micca tree^77^. Sampling heterogeneity was reduced rarefying samples at the depth of the less abundant sample (18,707 sequences).

Alpha- (number of observed OTUs and Shannon index) and beta- (weighed Unifrac and Bray–Curtis distances) diversity estimates were computed using the phyloseq R package^78^. Permutational MANOVA (PERMANOVA) was performed by using the adonis function of the vegan R package with 999 permutations. The identification of taxa differentially distributed in the groups of study was obtained by using the linear discriminant effect size analysis (LEfSe)^79^. Prediction of functional metabolic potential from metagenomic data was obtained by using Piphillin^46^ and the Kyoto Encyclopedia of Genes and Genomes (KEGG) October 2018 database^80^ with a 99% identity cut-off. All statistical analyses were performed using R^81^.

### Microbial isolation and phylogenetic characterization

Serial dilution method was used to count and isolate cultivable bacteria from coelomic fluid samples derived from individuals collected from both sites A and B. In particular, samples were serially diluted in sterile 0.45 µm-filtered sea water, 100 μl of each dilution was plated on marine agar medium (CONDA) and incubated for 24–48 h at 30 °C to allow bacterial growth. Then, a total of six isolates (namely A1-4 and B1-2 from A and B sites, respectively), each exhibiting representative colony morphology and/or pigmentation, were selected for phylogenetic analysis based on 16S rDNA sequence as previously described^82,83^. Briefly, 16S rDNA amplification was performed using the universal bacterial primers 27F and 1492R^84^. The PCR amplicons were purified according to the manufacturer’s protocol using PCR clean-up Gel extraction kit (Macherey–Nagel), and sequenced by Macrogen (http://www.macrogen.com/eng). The 16S rDNA sequences were reconstructed from raw forward and reverse sequence data by FinchTV software (Perkin Elmer) and analysed using the DECIPHER Find Chimeras web tool (http://decipher.cee.wisc.edu/FindChimeras.html). The six reconstructed bacterial 16S rDNA sequences were submitted to GenBank with the following accession numbers: MK492102 (A1); MK492110 (A2); MK492258 (A3); MK491620 (A4); MK492275 (B1); MK492272 (B2). These sequences were used as query for nucleotide BLAST interrogations against 16S ribosomal RNA sequences. The first ten hits for each query were selected, exported in FASTA format and then de-replicated. Then, a total of 48 16S rDNA sequences were used to perform a phylogenetic analysis by MEGA7^85^, using the Neighbor-Joining and Maximum Composite Likelihood methods to infer and compute the evolutionary history and distances, respectively^86^. All positions containing gaps and missing data were eliminated. There was a total of 866 positions in the final dataset. The 16S rDNA sequence of *Sulfolobus solfataricus* strain IFO 15331 was used as outgroup. Confidence for tree topologies was estimated by bootstrap values based on 1000 replicates.

### Preparation of bacterial samples and developmental toxicity assay

Overnight suspensions in Marine broth (CONDA) of individual bacterial strains isolated from coelomic fluid were split in two fractions. The first fraction was washed twice with MFSW by centrifugation at 6000 rpm for 10 min at 4 °C, and intact bacteria resuspended in MFSW. The bacterial pellet of the sister fraction was washed two times with phosphate buffered saline (PBS), resuspended in 500 μl of PBS and cells lysed by sonication using a Bandelin Sonopuls ultrasonic homogenizer. Following centrifugation at 12,000 rpm for 5 min, the supernatant containing hydrophilic substances was separated, while the pellet was incubated overnight with 0.5 M methanolic hydrochloric acid at room temperature, to extract lipophilic substances.

After gamete harvesting from fresh adult *P. lividus* echinoderms collected from the mentioned sites A and B, eggs were fertilized and cultured at 18 °C in MFSW, as previously described^87–89^. Individual bacterial strains from the above mentioned fraction were added to 24-well not-treated plates containing ~ 250 embryos/well at the concentration of 1 × 10^6^ and 1 × 10^7^/well. In parallel, sea urchin embryos from the same batches were exposed to bacterial lysate supernatants and methanolic extracts at the indicated amounts. Controls for this assay included sibling embryos exposed to equal amounts of PBS and methanolic hydrochloric acid solutions, respectively, as well as unperturbed embryos. Three replicates were reproduced for each experimental condition, and three independent experiments were performed using distinct embryo batches. Phenotypes of 100 embryos at the desired stage were examined under a Leica DM-4500B microscope, and digital images were processed using Adobe Photoshop CS10.

## Supplementary information

Supplementary information.

## Data Availability

The NGS datasets generated during and analysed during the current study are available in the European Nucleotide Archive repository (https://www.ebi.ac.uk/ena) with the accession identifier PRJEB27034. 16S rDNA sequences generated from the isolated bacterial strains are available in GenBank (https://www.ncbi.nlm.nih.gov/genbank) with the following accession numbers: MK492102 (A1); MK492110 (A2); MK492258 (A3); MK491620 (A4); MK492275 (B1); MK492272 (B2).
