# Supplementary material for: Composition and geographic variation of the bacterial microbiota associated with the coelomic fluid of the sea urchin Paracentrotus lividus
Source: Sci Rep. 2020 Dec 8;10:21443. doi: 10.1038/s41598-020-78534-5 (PMC7723044; doi:10.1038/s41598-020-78534-5)
Supplement: Supplementary file 1 — Supplementary information. [file 41598_2020_78534_MOESM1_ESM.pdf]

Supplementary material

Composition and Geographic Variation of the Bacterial Microbiota Associated with the Coelomic Fluid of the Sea Urchin *Paracentrotus lividus*

Teresa Faddetta[1#], Francesco Ardizzone[1#], Francesca Faillaci[1#], Chiara Reina[2], Emilia Palazzotto[1], Francesco Strati[3], Carlotta De Filippo[4], Giovanni Spinelli[1], Anna Maria Puglia[1], Giuseppe Gallo[1] and Vincenzo Cavalieri[1*]

[1] Department of Biological, Chemical and Pharmaceutical Sciences and Technologies, University of Palermo, viale delle Scienze, ed. 16, 90128 Palermo, Italy

[2] Department of Health Promotion, Mother and Child Care, Internal Medicine and Medical Specialties (PROMISE), University of Palermo, Piazza Delle Cliniche 2, 90127 Palermo, Italy

[3] Department of Experimental Oncology, European Institute of Oncology, Via Adamello, 16, 20139 Milano, Italy

[4] Institute of Agricultural Biology and Biotechnology, National Research Council, Via Moruzzi, 1, 56124 Pisa, Italy

[#] these authors contributed equally

*** Corresponding author**

E-mail: vincenzo.cavalieri@unipa.it

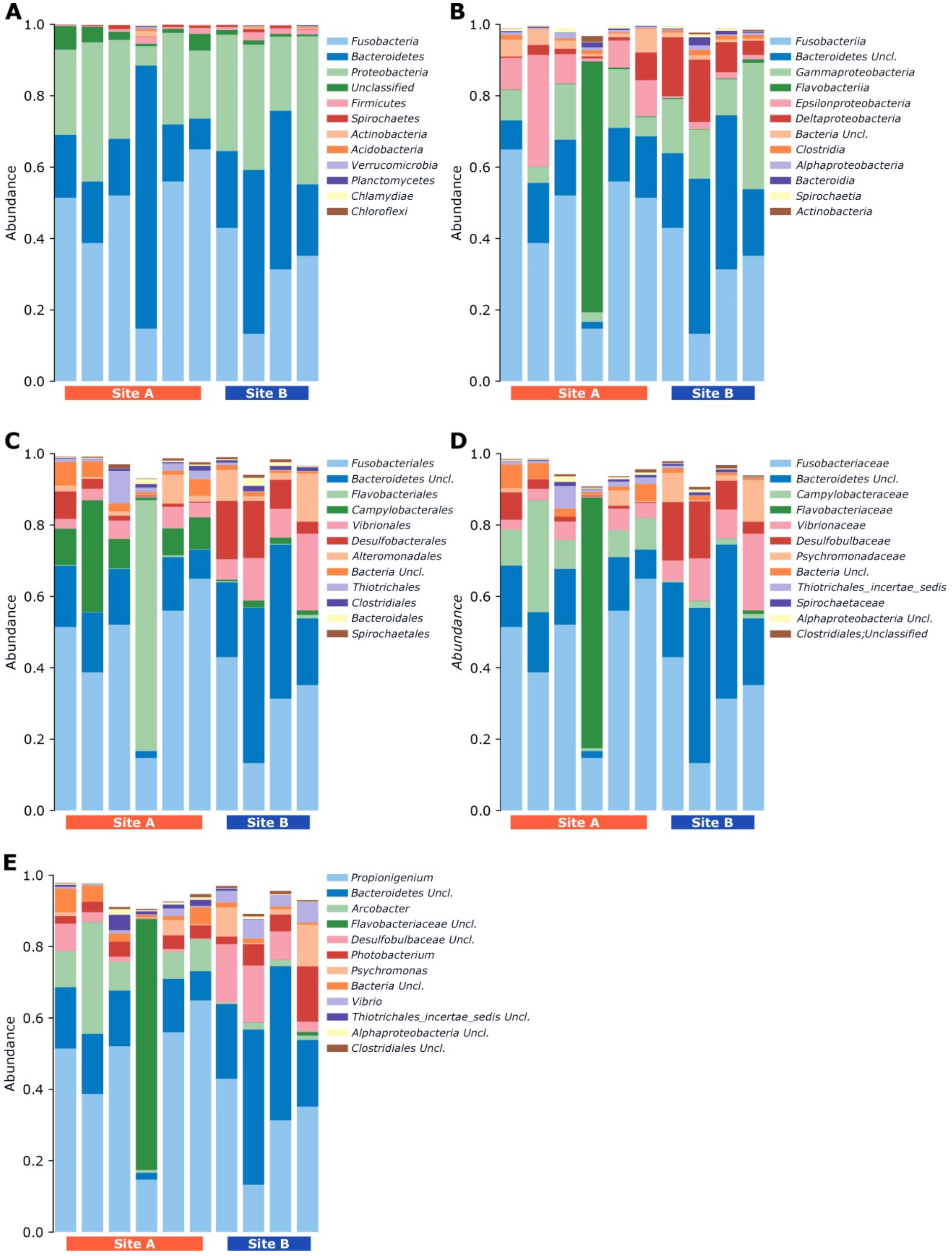

Figure S1. Relative abundances at A) phylum, B) class, C) order, D) family and E) genus level of *P. lividus* coelomic microbiota from sites A and B. T The top 12 most abundant taxa *per* taxonomic level are plotted.

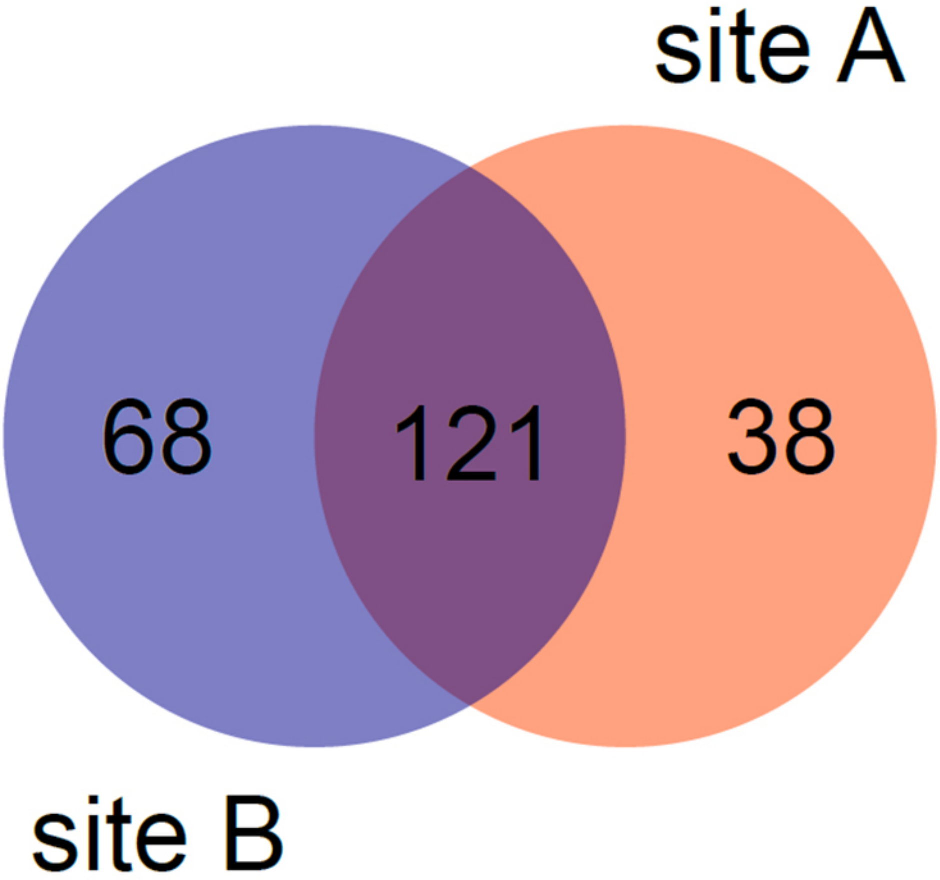

Figure S2. Venn diagram of bacterial genera shared between the coelomic fluid microbiota of *P. lividus* specimen collected from A and B sites.

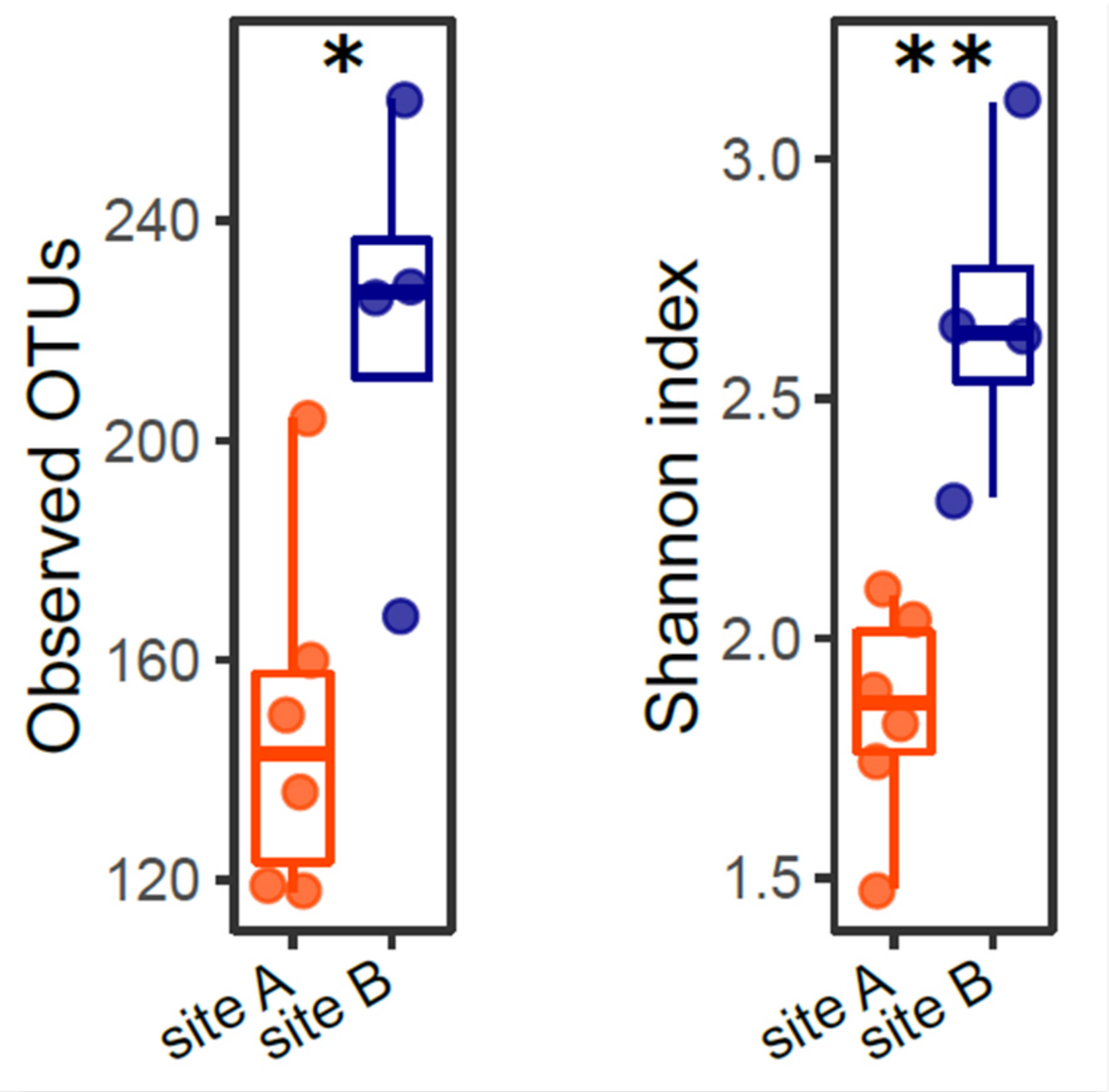

Figure S3. Alpha-diversity estimated on the observed number of OTUs and Shannon entropy index. The coelomic microbiota of *P. lividus* from the two sampling sites with different anthropogenic disturbance (site A vs site B) are coloured in orange and blue, respectively. *$p<0.05$, **$p<0.01$, Wilcoxon rank-sum test.

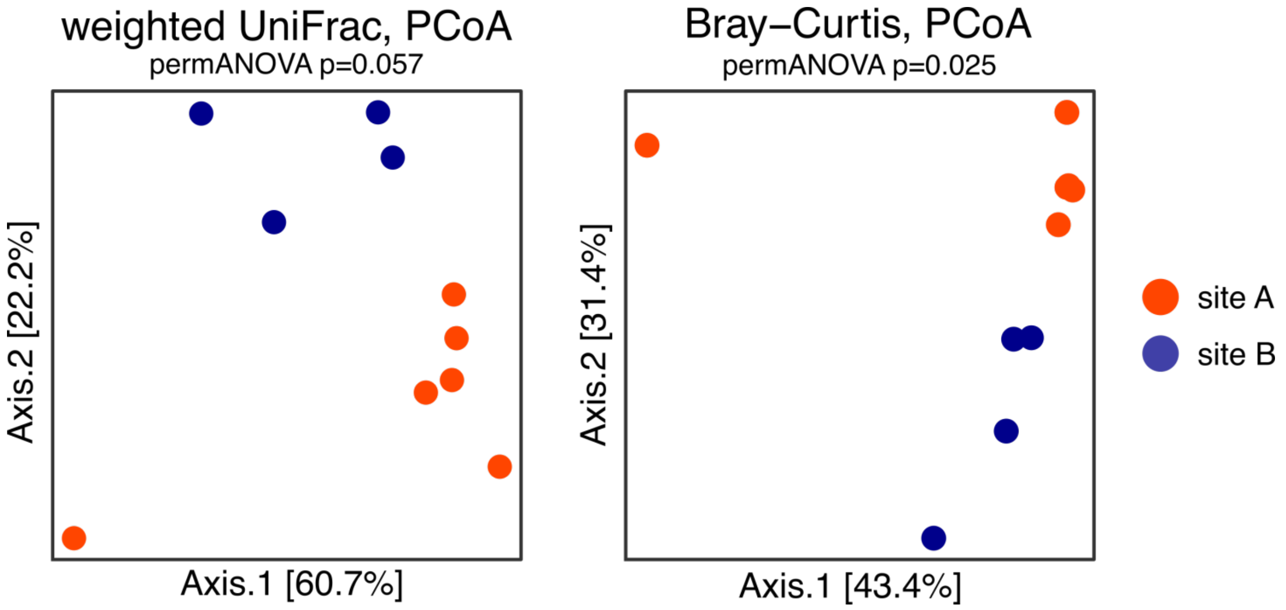

Figure S4. PCoA of bacterial beta-diversity based on the weighted UniFrac distance and Bray-Curtis dissimilarity. The exact *p*-value calculated on permutational multivariate analysis of variance (PERMANOVA) is shown.

Table S1. Taxonomical classification and abundance of bacterial microbiota members of *P. lividus* coelomic fluid based on NGS analysis of 16S rDNA v3-v4 region.

OTUs					B site %				A site %									
PHYLUM	CLASS	ORDER	FAMILY	GENUS	ID810-A1-1	ID810-A2-2	ID810-A3-3	ID810-A4-4	IID810-A5-5	ID810-A6-6	ID810-A7-7	ID810-A8-8	ID810-A9-9	ID810-A10-10	B % MEAN	A % MEAN	B % SD	A % SD
Acidobacteria	Acidobacteria_Gp10	Gp10	Gp10	Gp10	0,000	0,000	0,000	0,000	0,000	0,000	0,000	0,000	0,000	0,011	0,000	0,002	0,000	0,004
Acidobacteria	Acidobacteria_Gp17	Gp17	Gp17	Gp17	0,000	0,128	0,000	0,000	0,000	0,000	0,000	0,000	0,000	0,000	0,032	0,000	0,064	0,000
Acidobacteria	Acidobacteria_Gp25	Gp25	Gp25	Gp25	0,000	0,000	0,000	0,000	0,000	0,000	0,000	0,000	0,000	0,005	0,000	0,001	0,000	0,002
Acidobacteria	Acidobacteria_Gp3	Gp3	Gp3	Gp3	0,000	0,032	0,000	0,053	0,000	0,000	0,000	0,011	0,000	0,000	0,021	0,002	0,026	0,004
Acidobacteria	Acidobacteria_Gp4	Gp4	Gp4	Gp4	0,000	0,048	0,000	0,053	0,000	0,000	0,000	0,139	0,000	0,021	0,025	0,027	0,029	0,056
Acidobacteria	Acidobacteria_Gp5	Gp5	Gp5	Gp5	0,000	0,000	0,000	0,000	0,000	0,000	0,000	0,032	0,000	0,000	0,000	0,005	0,000	0,013
Acidobacteria	Acidobacteria_Gp6	Gp6	Gp6	Gp6	0,000	0,069	0,005	0,176	0,000	0,000	0,005	0,337	0,000	0,027	0,063	0,061	0,082	0,135
Acidobacteria	Acidobacteria_Gp7	Gp7	Gp7	Gp7	0,000	0,011	0,000	0,005	0,005	0,000	0,000	0,059	0,000	0,000	0,004	0,011	0,005	0,024
Acidobacteria	Acidobacteria_Gp1	Unclassified	Unclassified	Unclassified	0,000	0,000	0,000	0,000	0,000	0,000	0,000	0,000	0,000	0,005	0,000	0,001	0,000	0,002
Actinobacteria	Actinobacteria	Actinomycetales	Actinomycetaceae	Actinomyces	0,000	0,000	0,000	0,000	0,000	0,000	0,000	0,037	0,000	0,000	0,000	0,006	0,000	0,015
Actinobacteria	Actinobacteria	Actinomycetales	Microbacteriaceae	Agromyces	0,000	0,000	0,000	0,000	0,000	0,000	0,000	0,043	0,000	0,000	0,000	0,007	0,000	0,017
Actinobacteria	Actinobacteria	Actinomycetales	Micrococcaceae	Arthrobacter	0,005	0,000	0,000	0,011	0,000	0,000	0,000	0,000	0,000	0,000	0,004	0,000	0,005	0,000
Actinobacteria	Actinobacteria	Bifidobacteriales	Bifidobacteriaceae	Bifidobacterium	0,000	0,000	0,000	0,021	0,000	0,000	0,000	0,000	0,000	0,000	0,005	0,000	0,011	0,000
Actinobacteria	Actinobacteria	Coriobacteriales	Coriobacteriaceae	Collinsella	0,000	0,000	0,000	0,011	0,000	0,000	0,000	0,064	0,000	0,000	0,003	0,011	0,005	0,026
Actinobacteria	Actinobacteria	Actinomycetales	Corynebacteriaceae	Corynebacterium	0,000	0,016	0,000	0,027	0,000	0,000	0,000	0,000	0,000	0,000	0,011	0,000	0,013	0,000
Actinobacteria	Actinobacteria	Gaiellales	Gaiellaceae	Gaiella	0,000	0,000	0,000	0,032	0,000	0,000	0,000	0,064	0,000	0,000	0,008	0,011	0,016	0,026
Actinobacteria	Actinobacteria	Acidimicrobiales	Acidimicrobiaceae	Ilumatobacter	0,000	0,000	0,000	0,000	0,000	0,000	0,000	0,128	0,000	0,000	0,000	0,021	0,000	0,052
Actinobacteria	Actinobacteria	Actinomycetales	Nocardioidaceae	Nocardioides	0,000	0,000	0,000	0,000	0,000	0,000	0,000	0,000	0,000	0,011	0,000	0,002	0,000	0,004
Actinobacteria	Actinobacteria	Actinomycetales	Promicromonosporaceae	Promicromonospora	0,000	0,000	0,000	0,005	0,000	0,000	0,000	0,000	0,000	0,000	0,001	0,000	0,003	0,000
Actinobacteria	Actinobacteria	Actinomycetales	Propionibacteriaceae	Propionibacterium	0,016	0,374	0,021	0,230	0,000	0,032	0,000	0,957	0,005	0,037	0,160	0,172	0,174	0,385
Actinobacteria	Actinobacteria	Actinomycetales	Nocardiaceae	Rhodococcus	0,000	0,027	0,000	0,000	0,000	0,000	0,000	0,000	0,000	0,000	0,007	0,000	0,013	0,000
Actinobacteria	Actinobacteria	Actinomycetales	Micrococcaceae	Rothia	0,000	0,021	0,000	0,000	0,000	0,000	0,000	0,096	0,000	0,000	0,005	0,016	0,011	0,039
Actinobacteria	Actinobacteria	Rubrobacterales	Rubrobacteraceae	Rubrobacter	0,000	0,000	0,000	0,000	0,000	0,000	0,000	0,000	0,000	0,005	0,000	0,001	0,000	0,002
Actinobacteria	Actinobacteria	Actinomycetales	Streptomycetaceae	Streptomyces	0,000	0,064	0,005	0,000	0,005	0,005	0,000	0,075	0,011	0,043	0,017	0,023	0,031	0,030
Actinobacteria	Actinobacteria	Actinomycetales	Unclassified	Unclassified	0,011	0,000	0,005	0,032	0,000	0,000	0,000	0,203	0,005	0,000	0,012	0,035	0,014	0,083
Actinobacteria	Actinobacteria	Actinomycetales	Pseudonocardiaceae	Unclassified	0,000	0,000	0,000	0,048	0,000	0,000	0,000	0,000	0,000	0,000	0,012	0,000	0,024	0,000
Actinobacteria	Actinobacteria	Actinomycetales	Micromonosporaceae	Virgisporangium	0,000	0,011	0,000	0,000	0,000	0,000	0,000	0,000	0,000	0,000	0,003	0,000	0,005	0,000

Armatimonadetes	Armatimonadetes_gp2	Armatimonadetes_gp2	Armatimonadetes_gp2	Armatimonadetes_gp2	0,000	0,000	0,000	0,000	0,000	0,000	0,000	0,053	0,000	0,000	0,000	0,009	0,000	0,022
Armatimonadetes	Chthonomonadetes	Chthonomonadales	Chthonomonadaceae	Chthonomonas/Armatimonadetes_gp3	0,000	0,000	0,000	0,011	0,000	0,000	0,000	0,000	0,000	0,000	0,003	0,000	0,005	0,000
Bacteroidetes	Bacteroidia	Bacteroidales	Rikenellaceae	Alistipes	0,005	0,198	0,016	0,021	0,000	0,021	0,000	0,262	0,000	0,000	0,060	0,047	0,092	0,106
Bacteroidetes	Bacteroidia	Bacteroidales	Prevotellaceae	Alloprevotella	0,000	0,000	0,000	0,000	0,000	0,000	0,000	0,000	0,000	0,005	0,000	0,001	0,000	0,002
Bacteroidetes	Flavobacteriia	Flavobacteriales	Flavobacteriaceae	Arenibacter	0,000	0,000	0,000	0,005	0,000	0,000	0,000	0,000	0,000	0,000	0,001	0,000	0,003	0,000
Bacteroidetes	Bacteroidia	Bacteroidales	Bacteroidaceae	Bacteroides	0,005	0,150	0,016	0,102	0,000	0,000	0,000	0,454	0,016	0,027	0,068	0,083	0,069	0,182
Bacteroidetes	Bacteroidia	Bacteroidales	Porphyromonadaceae	Barnesiella	0,000	0,032	0,011	0,000	0,000	0,000	0,000	0,064	0,000	0,000	0,011	0,011	0,015	0,026
Bacteroidetes	Sphingobacteriia	Sphingobacteriales	Chitinophagaceae	Chitinophaga	0,000	0,021	0,000	0,000	0,000	0,000	0,000	0,000	0,000	0,000	0,005	0,000	0,011	0,000
Bacteroidetes	Flavobacteriia	Flavobacteriales	Flavobacteriaceae	Chryseobacterium	0,000	0,005	0,000	0,000	0,000	0,000	0,000	0,000	0,000	0,000	0,001	0,000	0,003	0,000
Bacteroidetes	Bacteroidia	Bacteroidales	Prolixibacteraceae	Draconibacterium	0,000	0,011	0,000	0,000	0,000	0,000	0,011	0,000	0,000	0,000	0,003	0,002	0,005	0,004
Bacteroidetes	Flavobacteriia	Flavobacteriales	Flavobacteriaceae	Lutibacter	0,000	0,000	0,000	0,000	0,000	0,005	0,021	0,000	0,005	0,000	0,000	0,005	0,000	0,008
Bacteroidetes	Bacteroidia	Bacteroidales	Porphyromonadaceae	Odoribacter	0,000	0,037	0,000	0,000	0,000	0,000	0,000	0,043	0,000	0,000	0,009	0,007	0,019	0,017
Bacteroidetes	Bacteroidetes_incertae_sedis	Ohtaekwangia	Ohtaekwangia	Ohtaekwangia	0,005	0,043	0,000	0,032	0,000	0,000	0,000	0,000	0,000	0,000	0,020	0,000	0,021	0,000
Bacteroidetes	Bacteroidia	Bacteroidales	Porphyromonadaceae	Parabacteroides	0,000	0,027	0,000	0,011	0,000	0,000	0,000	0,064	0,000	0,005	0,009	0,012	0,013	0,026
Bacteroidetes	Sphingobacteriia	Sphingobacteriales	Sphingobacteriaceae	Pedobacter	0,000	0,000	0,005	0,005	0,000	0,000	0,000	0,102	0,000	0,011	0,003	0,019	0,003	0,041
Bacteroidetes	Bacteroidia	Bacteroidales	Porphyromonadaceae	Porphyromonas	0,000	0,016	0,000	0,000	0,000	0,000	0,000	0,075	0,000	0,000	0,004	0,012	0,008	0,031
Bacteroidetes	Bacteroidia	Bacteroidales	Prevotellaceae	Prevotella	0,027	0,433	0,043	0,112	0,000	0,021	0,011	0,433	0,000	0,118	0,154	0,097	0,190	0,170
Bacteroidetes	Bacteroidia	Bacteroidales	Prolixibacteraceae	Sunxiuqinia	0,005	0,091	0,016	0,000	0,000	0,000	0,000	0,000	0,000	0,000	0,028	0,000	0,042	0,000
Bacteroidetes	Unclassified	Unclassified	Unclassified	Unclassified	20,949	43,486	43,171	18,672	17,202	16,860	15,657	1,919	15,026	8,179	31,570	12,474	13,610	6,133
Bacteroidetes	Flavobacteriia	Flavobacteriales	Flavobacteriaceae	Unclassified	0,000	0,075	0,048	0,994	0,005	0,053	0,016	70,311	0,005	0,037	0,279	11,738	0,478	28,695
Bacteroidetes	Bacteroidia	Bacteroidales	Unclassified	Unclassified	0,155	0,813	0,428	0,005	0,219	0,257	0,032	0,016	0,396	0,144	0,350	0,177	0,354	0,144
Bacteroidetes	Flavobacteriia	Flavobacteriales	Unclassified	Unclassified	0,331	0,000	0,182	0,005	0,225	0,027	0,134	0,000	0,492	0,080	0,130	0,159	0,159	0,181
Bacteroidetes	Bacteroidia	Bacteroidales	Prolixibacteraceae	Unclassified	0,080	0,449	0,513	0,021	0,005	0,000	0,005	0,000	0,016	0,053	0,266	0,013	0,251	0,020
Bacteroidetes	Sphingobacteriia	Sphingobacteriales	Chitinophagaceae	Unclassified	0,000	0,032	0,000	0,016	0,000	0,000	0,000	0,000	0,000	0,000	0,012	0,000	0,015	0,000
Bacteroidetes	Bacteroidia	Bacteroidales	Porphyromonadaceae	Unclassified	0,000	0,000	0,000	0,027	0,000	0,000	0,000	0,000	0,000	0,000	0,007	0,000	0,013	0,000
Bacteroidetes	Cytophagia	Cytophagales	Cytophagaceae	Unclassified	0,000	0,000	0,000	0,005	0,000	0,000	0,000	0,000	0,000	0,000	0,001	0,000	0,003	0,000
candidate division WPS-1	WPS-1_genera_incertae_sedis	WPS-1_genera_incertae_sedis	WPS-1_genera_incertae_sedis	WPS-1_genera_incertae_sedis	0,000	0,000	0,011	0,005	0,000	0,000	0,000	0,000	0,000	0,000	0,004	0,000	0,005	0,000
candidate division WPS-2	WPS-2_genera_incertae_sedis	WPS-2_genera_incertae_sedis	WPS-2_genera_incertae_sedis	WPS-2_genera_incertae_sedis	0,011	0,000	0,000	0,000	0,000	0,011	0,000	0,086	0,000	0,005	0,003	0,017	0,005	0,034
Candidatus Saccharibacteria	Saccharibacteria_genera_incertae_sedis	Saccharibacteria_genera_incertae_sedis	Saccharibacteria_genera_incertae_sedis	Saccharibacteria_genera_incertae_sedis	0,000	0,000	0,000	0,027	0,000	0,000	0,000	0,102	0,000	0,005	0,007	0,018	0,013	0,041
Chlamydiae	Chlamydiia	Chlamydiales	Simkaniaceae	Simkania	0,021	0,000	0,086	0,000	0,000	0,011	0,043	0,000	0,000	0,032	0,027	0,014	0,040	0,019
Chlamydiae	Chlamydiia	Chlamydiales	Unclassified	Unclassified	0,000	0,000	0,000	0,000	0,027	0,021	0,102	0,000	0,021	0,043	0,000	0,036	0,000	0,035
Chloroflexi	Unclassified	Unclassified	Unclassified	Unclassified	0,000	0,000	0,000	0,032	0,000	0,000	0,000	0,225	0,000	0,000	0,008	0,037	0,016	0,092

Chloroflexi	Anaerolineae	Anaerolineales	Anaerolineaceae	Unclassified	0,000	0,096	0,000	0,000	0,000	0,000	0,011	0,000	0,000	0,000	0,024	0,002	0,048	0,004
Cyanobacteria/Chloroplast	Chloroplast	Chloroplast	Chloroplast	Bacillariophyta	0,000	0,000	0,000	0,000	0,000	0,005	0,011	0,000	0,011	0,000	0,000	0,004	0,000	0,005
Cyanobacteria/Chloroplast	Chloroplast	Chloroplast	Chloroplast	Chlorophyta	0,000	0,000	0,000	0,011	0,000	0,000	0,000	0,000	0,000	0,000	0,003	0,000	0,005	0,000
Cyanobacteria/Chloroplast	Cyanobacteria	Family II	Family II	GpIIa	0,011	0,000	0,005	0,005	0,000	0,000	0,005	0,000	0,000	0,000	0,005	0,001	0,004	0,002
Cyanobacteria/Chloroplast	Cyanobacteria	Family VIII	Family VIII	GpVIII	0,000	0,000	0,000	0,000	0,000	0,000	0,000	0,000	0,000	0,005	0,000	0,001	0,000	0,002
Cyanobacteria/Chloroplast	Chloroplast	Chloroplast	Chloroplast	Streptophyta	0,000	0,005	0,000	0,000	0,005	0,053	0,005	0,000	0,011	0,000	0,001	0,012	0,003	0,020
Deferribacteres	Deferribacteres	Deferribacterales	Deferribacteraceae	Unclassified	0,000	0,021	0,000	0,000	0,000	0,000	0,000	0,000	0,000	0,000	0,005	0,000	0,011	0,000
Deinococcus-Thermus	Deinococci	Thermales	Thermaceae	Meiothermus	0,000	0,011	0,000	0,000	0,000	0,000	0,000	0,000	0,000	0,000	0,003	0,000	0,005	0,000
Fibrobacteres	Chitinivibrionia	Chitinivibrionales	Chitinivibrionaceae	Chitinivibrio	0,032	0,032	0,027	0,000	0,000	0,011	0,016	0,000	0,016	0,011	0,023	0,009	0,015	0,007
Firmicutes	Bacilli	Lactobacillales	Aerococcaceae	Aerococcus	0,000	0,011	0,000	0,000	0,000	0,005	0,000	0,000	0,000	0,000	0,003	0,001	0,005	0,002
Firmicutes	Clostridia	Clostridiales	Clostridiales_Incertae Sedis XI	Anaerococcus	0,005	0,000	0,000	0,005	0,000	0,000	0,000	0,000	0,005	0,005	0,003	0,002	0,003	0,003
Firmicutes	Clostridia	Clostridiales	Lachnospiraceae	Blautia	0,000	0,000	0,000	0,000	0,000	0,000	0,000	0,059	0,000	0,000	0,000	0,010	0,000	0,024
Firmicutes	Clostridia	Clostridiales	Ruminococcaceae	Clostridium IV	0,000	0,043	0,000	0,000	0,000	0,000	0,000	0,000	0,000	0,000	0,011	0,000	0,021	0,000
Firmicutes	Clostridia	Clostridiales	Clostridiaceae 1	Clostridium sensu stricto	0,000	0,000	0,000	0,011	0,000	0,000	0,000	0,000	0,000	0,000	0,003	0,000	0,005	0,000
Firmicutes	Clostridia	Clostridiales	Peptostreptococcaceae	Clostridium XI	0,000	0,032	0,000	0,000	0,000	0,000	0,000	0,000	0,000	0,000	0,008	0,000	0,016	0,000
Firmicutes	Clostridia	Clostridiales	Lachnospiraceae	Clostridium XlVa	0,000	0,043	0,016	0,064	0,000	0,000	0,000	0,064	0,000	0,005	0,031	0,012	0,028	0,026
Firmicutes	Erysipelotrichia	Erysipelotrichales	Erysipelotrichaceae	Clostridium XVIII	0,000	0,000	0,000	0,000	0,000	0,000	0,000	0,053	0,000	0,000	0,000	0,009	0,000	0,022
Firmicutes	Clostridia	Clostridiales	Lachnospiraceae	Coprococcus	0,000	0,000	0,000	0,000	0,000	0,000	0,000	0,000	0,000	0,011	0,000	0,002	0,000	0,004
Firmicutes	Bacilli	Lactobacillales	Carnobacteriaceae	Desemzia	0,000	0,000	0,016	0,000	0,000	0,000	0,000	0,000	0,000	0,000	0,004	0,000	0,008	0,000
Firmicutes	Negativicutes	Selenomonadales	Veillonellaceae	Dialister	0,005	0,037	0,000	0,000	0,000	0,005	0,000	0,000	0,000	0,000	0,011	0,001	0,018	0,002
Firmicutes	Clostridia	Clostridiales	Ruminococcaceae	Faecalibacterium	0,021	0,069	0,005	0,048	0,000	0,000	0,000	0,059	0,000	0,037	0,036	0,016	0,028	0,026
Firmicutes	Clostridia	Clostridiales	Clostridiales_Incertae Sedis XI	Finegoldia	0,000	0,000	0,000	0,011	0,000	0,000	0,000	0,000	0,000	0,000	0,003	0,000	0,005	0,000
Firmicutes	Clostridia	Clostridiales	Lachnospiraceae	Fusicatenibacter	0,000	0,000	0,000	0,000	0,000	0,000	0,000	0,043	0,000	0,000	0,000	0,007	0,000	0,017
Firmicutes	Bacilli	Bacillales	Bacillales_Incertae Sedis XI	Gemella	0,000	0,000	0,000	0,000	0,000	0,000	0,000	0,000	0,005	0,011	0,000	0,003	0,000	0,004
Firmicutes	Clostridia	Clostridiales	Ruminococcaceae	Gemmiger	0,000	0,021	0,005	0,027	0,000	0,011	0,000	0,075	0,000	0,000	0,013	0,014	0,013	0,030
Firmicutes	Clostridia	Clostridiales	Lachnospiraceae	Lachnospiracea_incertae_sedis	0,000	0,064	0,005	0,037	0,000	0,000	0,000	0,000	0,000	0,000	0,027	0,000	0,030	0,000
Firmicutes	Bacilli	Lactobacillales	Lactobacillaceae	Lactobacillus	0,005	0,069	0,000	0,032	0,000	0,005	0,000	0,091	0,000	0,000	0,027	0,016	0,032	0,037
Firmicutes	Bacilli	Bacillales	Planococcaceae	Lysinibacillus	0,011	0,059	0,000	0,059	0,000	0,000	0,000	0,289	0,005	0,005	0,032	0,050	0,031	0,117
Firmicutes	Clostridia	Clostridiales	Natranaerovirga	Natranaerovirga	0,069	0,176	0,075	0,139	0,005	0,000	0,005	0,000	0,048	0,198	0,115	0,043	0,052	0,078
Firmicutes	Clostridia	Clostridiales	Ruminococcaceae	Oscillibacter	0,000	0,037	0,000	0,027	0,000	0,000	0,000	0,000	0,000	0,000	0,016	0,000	0,019	0,000
Firmicutes	Clostridia	Clostridiales	Clostridiales_Incertae Sedis XI	Peptoniphilus	0,000	0,000	0,000	0,000	0,000	0,000	0,000	0,000	0,005	0,000	0,000	0,001	0,000	0,002
Firmicutes	Negativicutes	Selenomonadales	Acidaminococcaceae	Phascolarctobacterium	0,000	0,000	0,000	0,011	0,000	0,000	0,000	0,000	0,000	0,000	0,003	0,000	0,005	0,000

Firmicutes	Clostridia	Clostridiales	Lachnospiraceae	Roseburia	0,000	0,011	0,000	0,011	0,000	0,000	0,000	0,000	0,000	0,005	0,005	0,001	0,006	0,002
Firmicutes	Clostridia	Clostridiales	Ruminococcaceae	Ruminococcus	0,000	0,000	0,011	0,021	0,000	0,000	0,000	0,069	0,000	0,005	0,008	0,012	0,010	0,028
Firmicutes	Bacilli	Bacillales	Staphylococcaceae	Staphylococcus	0,000	0,112	0,043	0,037	0,000	0,016	0,000	0,144	0,027	0,021	0,048	0,035	0,047	0,055
Firmicutes	Bacilli	Lactobacillales	Streptococcaceae	Streptococcus	0,000	0,000	0,000	0,000	0,000	0,000	0,000	0,048	0,005	0,032	0,000	0,014	0,000	0,021
Firmicutes	Bacilli	Lactobacillales	Carnobacteriaceae	Trichococcus	0,000	0,005	0,000	0,000	0,000	0,000	0,000	0,000	0,000	0,000	0,001	0,000	0,003	0,000
Firmicutes	Clostridia	Clostridiales	Unclassified	Unclassified	0,567	0,732	0,925	0,385	0,214	0,118	0,588	0,385	0,251	0,903	0,652	0,410	0,231	0,292
Firmicutes	Unclassified	Unclassified	Unclassified	Unclassified	0,150	0,369	0,390	0,128	0,032	0,048	0,091	0,118	0,128	0,214	0,259	0,105	0,139	0,065
Firmicutes	Clostridia	Clostridiales	Ruminococcaceae	Unclassified	0,005	0,150	0,037	0,171	0,005	0,011	0,043	0,102	0,021	0,155	0,091	0,056	0,082	0,060
Firmicutes	Clostridia	Clostridiales	Lachnospiraceae	Unclassified	0,000	0,144	0,037	0,000	0,000	0,000	0,000	0,144	0,005	0,016	0,045	0,028	0,068	0,058
Firmicutes	Clostridia	Clostridiales	Eubacteriaceae	Unclassified	0,000	0,000	0,005	0,069	0,005	0,000	0,000	0,000	0,000	0,000	0,019	0,001	0,034	0,002
Firmicutes	Bacilli	Bacillales	Planococcaceae	Unclassified	0,000	0,048	0,000	0,000	0,000	0,000	0,000	0,000	0,000	0,000	0,012	0,000	0,024	0,000
Firmicutes	Negativicutes	Selenomonadales	Veillonellaceae	Veillonella	0,000	0,000	0,000	0,011	0,000	0,000	0,000	0,059	0,000	0,000	0,003	0,010	0,005	0,024
Firmicutes	Bacilli	Bacillales	Bacillaceae 2	Virgibacillus	0,000	0,000	0,000	0,000	0,000	0,000	0,000	0,000	0,011	0,000	0,000	0,002	0,000	0,004
Fusobacteria	Fusobacteriia	Fusobacteriales	Fusobacteriaceae	Propionigenium	42,963	13,289	31,347	35,158	51,414	38,718	52,055	14,727	55,995	64,933	30,689	46,307	12,567	17,634
Gemmatimonadetes	Gemmatimonadetes	Gemmatimonadales	Gemmatimonadaceae	Gemmatimonas	0,000	0,000	0,000	0,064	0,000	0,000	0,000	0,032	0,000	0,000	0,016	0,005	0,032	0,013
Latescibacteria	Latescibacteria_genera_incertae_sedis	Latescibacteria_genera_incertae_sedis	Latescibacteria_genera_incertae_sedis	Latescibacteria_genera_incertae_sedis	0,000	0,000	0,000	0,011	0,000	0,000	0,000	0,000	0,000	0,000	0,003	0,000	0,005	0,000
Nitrospirae	Nitrospira	Nitrospirales	Nitrospiraceae	Nitrospira	0,000	0,000	0,000	0,000	0,000	0,000	0,000	0,000	0,000	0,021	0,000	0,004	0,000	0,009
Parcubacteria	Parcubacteria_genera_incertae_sedis	Parcubacteria_genera_incertae_sedis	Parcubacteria_genera_incertae_sedis	Parcubacteria_genera_incertae_sedis	0,000	0,000	0,000	0,032	0,000	0,000	0,000	0,102	0,000	0,021	0,008	0,020	0,016	0,041
Planctomycetes	Planctomycetia	Planctomycetales	Planctomycetaceae	Gemmata	0,000	0,000	0,000	0,075	0,000	0,000	0,000	0,000	0,005	0,000	0,019	0,001	0,037	0,002
Planctomycetes	Planctomycetia	Planctomycetales	Planctomycetaceae	Pirellula	0,000	0,000	0,000	0,037	0,005	0,000	0,000	0,139	0,000	0,005	0,009	0,025	0,019	0,056
Planctomycetes	Planctomycetia	Planctomycetales	Planctomycetaceae	Unclassified	0,000	0,064	0,011	0,107	0,000	0,000	0,000	0,048	0,005	0,000	0,045	0,009	0,050	0,019
Planctomycetes	Planctomycetia	Unclassified	Unclassified	Unclassified	0,000	0,000	0,000	0,011	0,000	0,000	0,000	0,000	0,000	0,000	0,003	0,000	0,005	0,000
Proteobacteria	Gammaproteobacteria	Pseudomonadales	Moraxellaceae	Acinetobacter	0,000	0,011	0,005	0,000	0,000	0,000	0,000	0,016	0,000	0,000	0,004	0,003	0,005	0,007
Proteobacteria	Gammaproteobacteria	Alteromonadales	Alteromonadaceae	Agarivorans	0,080	0,000	0,037	0,059	0,005	0,000	0,021	0,000	0,016	0,000	0,044	0,007	0,034	0,009
Proteobacteria	Gammaproteobacteria	Oceanospirillales	Alcanivoracaceae	Alcanivorax	0,000	0,011	0,000	0,005	0,000	0,000	0,000	0,000	0,000	0,000	0,004	0,000	0,005	0,000
Proteobacteria	Gammaproteobacteria	Vibrionales	Vibrionaceae	Aliivibrio	0,011	0,401	0,107	0,037	0,005	0,000	0,000	0,000	0,000	0,000	0,139	0,001	0,179	0,002
Proteobacteria	Alphaproteobacteria	Rhodobacterales	Rhodobacteraceae	Amaricoccus	0,000	0,043	0,000	0,000	0,000	0,000	0,000	0,000	0,000	0,000	0,011	0,000	0,021	0,000
Proteobacteria	Deltaproteobacteria	Myxococcales	Cystobacteraceae	Anaeromyxobacter	0,000	0,000	0,000	0,032	0,000	0,000	0,000	0,086	0,000	0,000	0,008	0,014	0,016	0,035
Proteobacteria	Epsilonproteobacteria	Campylobacterales	Campylobacteraceae	Arcobacter	0,465	1,919	1,721	1,240	10,125	31,261	8,093	0,786	7,484	8,916	1,336	11,111	0,647	10,399
Proteobacteria	Gammaproteobacteria	Xanthomonadales	Xanthomonadaceae	Arenimonas	0,000	0,000	0,000	0,021	0,000	0,000	0,000	0,000	0,000	0,000	0,005	0,000	0,011	0,000
Proteobacteria	Deltaproteobacteria	Bdellovibrionales	Bacteriovoracaceae	Bacteriovorax	0,027	0,053	0,005	0,000	0,000	0,000	0,000	0,000	0,000	0,000	0,021	0,000	0,024	0,000
Proteobacteria	Gammaproteobacteria	Oceanospirillales	Oceanospirillaceae	Bermanella	0,027	0,091	0,011	0,000	0,000	0,000	0,000	0,000	0,000	0,000	0,032	0,000	0,041	0,000

Proteobacteria	Alphaproteobacteria	Rhizobiales	Bradyrhizobiaceae	Bradyrhizobium	0,005	0,080	0,005	0,048	0,000	0,000	0,000	0,064	0,000	0,000	0,035	0,011	0,036	0,026
Proteobacteria	Alphaproteobacteria	Caulobacterales	Caulobacteraceae	Brevundimonas	0,000	0,027	0,000	0,000	0,000	0,000	0,000	0,075	0,000	0,000	0,007	0,012	0,013	0,031
Proteobacteria	Gammaproteobacteria	Alteromonadales	Colwelliaceae	Colwellia	0,005	0,000	0,005	0,000	0,000	0,000	0,000	0,000	0,000	0,000	0,003	0,000	0,003	0,000
Proteobacteria	Gammaproteobacteria	Gammaproteobacteria_incertae_sedis	Congregibacter	Congregibacter	0,000	0,000	0,000	0,016	0,000	0,000	0,000	0,000	0,000	0,000	0,004	0,000	0,008	0,000
Proteobacteria	Betaproteobacteria	Burkholderiales	Comamonadaceae	Delftia	0,000	0,000	0,005	0,011	0,000	0,000	0,000	0,086	0,000	0,011	0,004	0,016	0,005	0,034
Proteobacteria	Deltaproteobacteria	Desulfobacterales	Desulfobulbaceae	Desulfotalea	0,048	0,032	0,064	0,470	0,005	0,000	0,000	0,123	0,059	0,064	0,154	0,042	0,212	0,049
Proteobacteria	Deltaproteobacteria	Desulfovibrionales	Desulfovibrionaceae	Desulfovibrio	0,053	1,026	0,112	0,294	0,000	0,011	0,005	0,000	0,000	0,053	0,372	0,012	0,448	0,021
Proteobacteria	Alphaproteobacteria	Rhodospirillales	Rhodospirillaceae	Dongia	0,000	0,000	0,000	0,027	0,000	0,000	0,005	0,075	0,000	0,000	0,007	0,013	0,013	0,030
Proteobacteria	Gammaproteobacteria	Oceanospirillales	Hahellaceae	Endozoicomonas	0,000	0,000	0,000	0,000	0,000	0,005	0,043	0,000	0,000	0,000	0,000	0,008	0,000	0,017
Proteobacteria	Gammaproteobacteria	Vibrionales	Vibrionaceae	Enterovibrio	0,011	0,005	0,005	0,000	0,000	0,000	0,000	0,000	0,000	0,000	0,005	0,000	0,004	0,000
Proteobacteria	Gammaproteobacteria	Enterobacteriales	Enterobacteriaceae	Erwinia	0,000	0,005	0,000	0,000	0,000	0,000	0,000	0,027	0,000	0,000	0,001	0,004	0,003	0,011
Proteobacteria	Gammaproteobacteria	Enterobacteriales	Enterobacteriaceae	Escherichia/Shigella	0,000	0,043	0,000	0,000	0,000	0,000	0,000	0,000	0,000	0,016	0,011	0,003	0,021	0,007
Proteobacteria	Gammaproteobacteria	Thiotrichales	Thiotrichales_incertae_sedis	Fangia	0,037	0,027	0,059	0,011	0,315	0,502	1,973	0,005	0,305	0,139	0,033	0,540	0,020	0,722
Proteobacteria	Gammaproteobacteria	Alteromonadales	Ferrimonadaceae	Ferrimonas	0,027	0,112	0,032	0,011	0,021	0,021	0,251	0,000	0,519	0,048	0,045	0,143	0,045	0,206
Proteobacteria	Gammaproteobacteria	Thiotrichales	Francisellaceae	Francisella	0,075	0,005	0,005	0,118	0,091	0,005	2,839	0,390	0,727	0,529	0,051	0,764	0,055	1,052
Proteobacteria	Deltaproteobacteria	Desulfuromonadales	Geobacteraceae	Geobacter	0,000	0,000	0,000	0,000	0,000	0,000	0,000	0,000	0,000	0,005	0,000	0,001	0,000	0,002
Proteobacteria	Gammaproteobacteria	Orbales	Orbaceae	Gilliamella	0,000	0,043	0,011	0,000	0,000	0,000	0,000	0,027	0,000	0,000	0,013	0,004	0,020	0,011
Proteobacteria	Gammaproteobacteria	Pasteurellales	Pasteurellaceae	Haemophilus	0,000	0,000	0,000	0,000	0,000	0,000	0,000	0,021	0,000	0,011	0,000	0,005	0,000	0,009
Proteobacteria	Epsilonproteobacteria	Campylobacterales	Helicobacteraceae	Helicobacter	0,000	0,075	0,000	0,000	0,000	0,000	0,000	0,000	0,000	0,000	0,019	0,000	0,037	0,000
Proteobacteria	Gammaproteobacteria	Alteromonadales	Idiomarinaceae	Idiomarina	0,000	0,021	0,000	0,000	0,000	0,000	0,000	0,000	0,000	0,000	0,005	0,000	0,011	0,000
Proteobacteria	Gammaproteobacteria	Oceanospirillales	Hahellaceae	Kistimonas	0,000	0,000	0,000	0,000	0,000	0,000	0,000	0,000	0,000	0,005	0,000	0,001	0,000	0,002
Proteobacteria	Deltaproteobacteria	Myxococcales	Kofleriaceae	Kofleria	0,000	0,000	0,011	0,000	0,000	0,000	0,005	0,000	0,000	0,000	0,003	0,001	0,005	0,002
Proteobacteria	Alphaproteobacteria	Rhodobacterales	Rhodobacteraceae	Labrenzia	0,000	0,000	0,000	0,011	0,000	0,000	0,000	0,000	0,000	0,000	0,003	0,000	0,005	0,000
Proteobacteria	Gammaproteobacteria	Legionellales	Legionellaceae	Legionella	0,000	0,000	0,000	0,037	0,000	0,000	0,000	0,000	0,000	0,000	0,009	0,000	0,019	0,000
Proteobacteria	Gammaproteobacteria	Alteromonadales	Alteromonadaceae	Marinobacter	0,000	0,032	0,000	0,000	0,000	0,000	0,000	0,000	0,000	0,000	0,008	0,000	0,016	0,000
Proteobacteria	Betaproteobacteria	Burkholderiales	Oxalobacteraceae	Massilia	0,005	0,000	0,000	0,027	0,000	0,000	0,000	0,000	0,000	0,000	0,008	0,000	0,013	0,000
Proteobacteria	Alphaproteobacteria	Rhizobiales	Phyllobacteriaceae	Mesorhizobium	0,000	0,032	0,000	0,000	0,000	0,000	0,000	0,000	0,000	0,005	0,008	0,001	0,016	0,002
Proteobacteria	Alphaproteobacteria	Rhizobiales	Methylobacteriaceae	Methylobacterium	0,000	0,005	0,000	0,000	0,000	0,000	0,000	0,000	0,000	0,000	0,001	0,000	0,003	0,000
Proteobacteria	Betaproteobacteria	Methylophilales	Methylophilaceae	Methylophilus	0,000	0,000	0,005	0,000	0,000	0,000	0,000	0,000	0,000	0,011	0,001	0,002	0,003	0,004
Proteobacteria	Alphaproteobacteria	Rhizobiales	Methylobacteriaceae	Microvirga	0,000	0,000	0,000	0,096	0,000	0,000	0,000	0,000	0,000	0,000	0,024	0,000	0,048	0,000
Proteobacteria	Gammaproteobacteria	Alteromonadales	Moritellaceae	Moritella	0,000	0,000	0,000	0,016	0,005	0,000	0,005	0,000	0,000	0,000	0,004	0,002	0,008	0,003
Proteobacteria	Gammaproteobacteria	Alteromonadales	Neiella	Neiella	0,075	0,000	0,000	0,000	0,000	0,000	0,000	0,000	0,000	0,000	0,019	0,000	0,037	0,000

Proteobacteria	Betaproteobacteria	Neisseriales	Neisseriaceae	Neisseria	0,000	0,005	0,000	0,000	0,000	0,000	0,000	0,000	0,000	0,027	0,001	0,004	0,003	0,011
Proteobacteria	Alphaproteobacteria	Rhodobacterales	Rhodobacteraceae	Paracoccus	0,011	0,000	0,000	0,000	0,000	0,000	0,000	0,032	0,000	0,000	0,003	0,005	0,005	0,013
Proteobacteria	Alphaproteobacteria	Rhodospirillales	Rhodospirillaceae	Pelagibius	0,000	0,000	0,000	0,005	0,000	0,000	0,005	0,000	0,000	0,000	0,001	0,001	0,003	0,002
Proteobacteria	Alphaproteobacteria	Rhodobacterales	Rhodobacteraceae	Phaeobacter	0,027	0,118	0,000	0,016	0,000	0,000	0,000	0,011	0,000	0,000	0,040	0,002	0,053	0,004
Proteobacteria	Alphaproteobacteria	Caulobacterales	Caulobacteraceae	Phenylobacterium	0,000	0,000	0,000	0,016	0,000	0,000	0,000	0,037	0,000	0,000	0,004	0,006	0,008	0,015
Proteobacteria	Gammaproteobacteria	Vibrionales	Vibrionaceae	Photobacterium	2,144	6,014	4,693	15,545	2,101	2,956	4,212	0,096	3,854	3,704	7,099	2,821	5,855	1,533
Proteobacteria	Alphaproteobacteria	Rhizobiales	Phyllobacteriaceae	Phyllobacterium	0,000	0,011	0,000	0,000	0,000	0,000	0,000	0,043	0,000	0,000	0,003	0,007	0,005	0,017
Proteobacteria	Betaproteobacteria	Rhodocyclales	Rhodocyclaceae	Propionivibrio	0,000	0,000	0,000	0,000	0,000	0,000	0,000	0,048	0,000	0,000	0,000	0,008	0,000	0,020
Proteobacteria	Gammaproteobacteria	Alteromonadales	Pseudoalteromonadaceae	Pseudoalteromonas	0,016	0,037	0,005	0,000	0,000	0,000	0,000	0,021	0,000	0,000	0,015	0,004	0,017	0,009
Proteobacteria	Gammaproteobacteria	Pseudomonadales	Pseudomonadaceae	Pseudomonas	0,000	0,021	0,005	0,021	0,000	0,000	0,000	0,032	0,000	0,000	0,012	0,005	0,011	0,013
Proteobacteria	Gammaproteobacteria	Pseudomonadales	Moraxellaceae	Psychrobacter	0,021	0,123	0,011	0,000	0,000	0,000	0,000	0,000	0,000	0,000	0,039	0,000	0,057	0,000
Proteobacteria	Gammaproteobacteria	Alteromonadales	Psychromonadaceae	Psychromonas	8,184	0,347	1,529	11,702	1,107	0,091	0,011	0,219	4,271	0,310	5,440	1,001	5,415	1,649
Proteobacteria	Gammaproteobacteria	Alteromonadales	Pseudoalteromonadaceae	Psychrosphaera	0,005	0,278	0,000	0,000	0,000	0,000	0,000	0,000	0,000	0,000	0,071	0,000	0,138	0,000
Proteobacteria	Betaproteobacteria	Burkholderiales	Burkholderiaceae	Ralstonia	0,000	0,000	0,000	0,021	0,000	0,000	0,000	0,000	0,000	0,000	0,005	0,000	0,011	0,000
Proteobacteria	Alphaproteobacteria	Rhizobiales	Rhizobiaceae	Rhizobium	0,000	0,000	0,000	0,027	0,000	0,016	0,000	0,198	0,000	0,000	0,007	0,036	0,013	0,080
Proteobacteria	Alphaproteobacteria	Rhodobacterales	Rhodobacteraceae	Ruegeria	0,027	0,037	0,000	0,021	0,000	0,000	0,011	0,000	0,000	0,000	0,021	0,002	0,016	0,004
Proteobacteria	Gammaproteobacteria	Oceanospirillales	Saccharospirillaceae	Saccharospirillum	0,011	0,000	0,000	0,000	0,000	0,000	0,000	0,000	0,000	0,000	0,003	0,000	0,005	0,000
Proteobacteria	Alphaproteobacteria	Rhizobiales	Bradyrhizobiaceae	Salinarimonas	0,000	0,011	0,000	0,000	0,000	0,000	0,000	0,000	0,000	0,000	0,003	0,000	0,005	0,000
Proteobacteria	Gammaproteobacteria	Enterobacteriales	Enterobacteriaceae	Serratia	0,000	0,000	0,000	0,021	0,000	0,000	0,000	0,000	0,000	0,000	0,005	0,000	0,011	0,000
Proteobacteria	Gammaproteobacteria	Alteromonadales	Shewanellaceae	Shewanella	0,102	0,225	0,032	0,166	0,123	0,069	0,406	0,000	0,679	0,171	0,131	0,241	0,083	0,255
Proteobacteria	Alphaproteobacteria	Sphingomonadales	Sphingomonadaceae	Sphingomonas	0,000	0,027	0,000	0,005	0,000	0,005	0,005	0,005	0,000	0,011	0,008	0,004	0,013	0,004
Proteobacteria	Gammaproteobacteria	Oceanospirillales	Oceanospirillales_incertae_sedis	Spongiispira	0,000	0,032	0,000	0,000	0,000	0,000	0,000	0,000	0,000	0,000	0,008	0,000	0,016	0,000
Proteobacteria	Epsilonproteobacteria	Campylobacterales	Helicobacteraceae	Sulfurimonas	0,000	0,000	0,000	0,000	0,011	0,000	0,134	0,000	0,064	0,016	0,000	0,037	0,000	0,053
Proteobacteria	Betaproteobacteria	Burkholderiales	Sutterellaceae	Sutterella	0,000	0,000	0,011	0,027	0,000	0,000	0,000	0,000	0,000	0,000	0,009	0,000	0,013	0,000
Proteobacteria	Deltaproteobacteria	Syntrophobacterales	Syntrophobacteraceae	Syntrophobacter	0,000	0,000	0,000	0,000	0,000	0,000	0,000	0,048	0,000	0,000	0,000	0,008	0,000	0,020
Proteobacteria	Gammaproteobacteria	Alteromonadales	Colwelliaceae	Thalassomonas	0,086	0,075	0,176	1,422	0,203	0,112	0,048	0,000	0,315	0,203	0,440	0,147	0,656	0,116
Proteobacteria	Deltaproteobacteria	Desulfobacterales	Desulfobulbaceae	Unclassified	16,304	15,908	7,992	2,876	7,692	2,753	1,379	0,198	0,796	0,198	10,770	2,169	6,508	2,868
Proteobacteria	Gammaproteobacteria	Thiotrichales	Thiotrichales_incertae_sedis	Unclassified	0,609	0,134	0,053	0,059	0,561	0,080	4,330	0,850	1,091	1,700	0,214	1,435	0,266	1,517
Proteobacteria	Gammaproteobacteria	Alteromonadales	Unclassified	Unclassified	0,086	0,166	0,016	0,102	0,166	0,225	0,417	0,037	2,299	0,834	0,092	0,663	0,061	0,848
Proteobacteria	Alphaproteobacteria	Unclassified	Unclassified	Unclassified	0,155	0,780	0,299	0,171	0,364	0,273	1,582	0,289	0,615	0,700	0,351	0,637	0,293	0,495
Proteobacteria	Unclassified	Unclassified	Unclassified	Unclassified	0,214	0,529	0,203	0,134	0,187	0,075	0,738	0,150	0,144	0,353	0,270	0,274	0,176	0,245
Proteobacteria	Gammaproteobacteria	Unclassified	Unclassified	Unclassified	0,016	0,053	0,021	0,032	0,064	0,187	0,086	0,401	0,144	0,337	0,031	0,203	0,017	0,137

Proteobacteria	Betaproteobacteria	Burkholderiales	Oxalobacteraceae	Unclassified	0,000	0,027	0,005	0,000	0,005	0,037	0,075	0,053	0,080	0,134	0,008	0,064	0,013	0,044
Proteobacteria	Gammaproteobacteria	Methylococcales	Methylococcaceae	Unclassified	0,000	0,000	0,000	0,000	0,000	0,000	0,000	0,337	0,000	0,000	0,000	0,056	0,000	0,137
Proteobacteria	Deltaproteobacteria	Myxococcales	Polyangiaceae	Unclassified	0,000	0,000	0,000	0,000	0,053	0,000	0,102	0,000	0,016	0,000	0,000	0,029	0,000	0,041
Proteobacteria	Gammaproteobacteria	Vibrionales	Vibrionaceae	Unclassified	0,203	0,166	0,171	0,219	0,139	0,000	0,005	0,000	0,016	0,005	0,190	0,028	0,026	0,055
Proteobacteria	Deltaproteobacteria	Myxococcales	Unclassified	Unclassified	0,000	0,064	0,000	0,091	0,000	0,000	0,000	0,144	0,000	0,000	0,039	0,024	0,046	0,059
Proteobacteria	Betaproteobacteria	Burkholderiales	Unclassified	Unclassified	0,000	0,000	0,000	0,000	0,005	0,000	0,000	0,091	0,000	0,000	0,000	0,016	0,000	0,037
Proteobacteria	Betaproteobacteria	Unclassified	Unclassified	Unclassified	0,000	0,000	0,000	0,000	0,000	0,000	0,000	0,080	0,000	0,000	0,000	0,013	0,000	0,033
Proteobacteria	Gammaproteobacteria	Xanthomonadales	Xanthomonadaceae	Unclassified	0,000	0,000	0,000	0,027	0,000	0,000	0,000	0,069	0,000	0,000	0,007	0,012	0,013	0,028
Proteobacteria	Deltaproteobacteria	Desulfovibrionales	Desulfovibrionaceae	Unclassified	0,005	0,000	0,000	0,000	0,000	0,005	0,016	0,000	0,005	0,043	0,001	0,012	0,003	0,016
Proteobacteria	Deltaproteobacteria	Desulfovibrionales	Unclassified	Unclassified	0,059	0,182	0,102	0,203	0,000	0,000	0,000	0,016	0,005	0,011	0,136	0,005	0,068	0,007
Proteobacteria	Deltaproteobacteria	Desulfobacterales	Desulfobacteraceae	Unclassified	0,000	0,091	0,096	0,000	0,000	0,011	0,000	0,000	0,011	0,000	0,047	0,004	0,054	0,006
Proteobacteria	Gammaproteobacteria	Alteromonadales	Colwelliaceae	Unclassified	0,011	0,000	0,027	0,043	0,000	0,005	0,000	0,000	0,000	0,016	0,020	0,004	0,019	0,006
Proteobacteria	Betaproteobacteria	Neisseriales	Neisseriaceae	Unclassified	0,000	0,037	0,000	0,000	0,000	0,000	0,000	0,000	0,005	0,011	0,009	0,003	0,019	0,004
Proteobacteria	Epsilonproteobacteria	Campylobacterales	Unclassified	Unclassified	0,000	0,000	0,000	0,000	0,000	0,000	0,000	0,000	0,000	0,011	0,000	0,002	0,000	0,004
Proteobacteria	Gammaproteobacteria	Pasteurellales	Pasteurellaceae	Unclassified	0,000	0,000	0,000	0,000	0,000	0,000	0,000	0,000	0,000	0,011	0,000	0,002	0,000	0,004
Proteobacteria	Deltaproteobacteria	Unclassified	Unclassified	Unclassified	0,000	0,128	0,011	0,027	0,000	0,000	0,000	0,000	0,000	0,005	0,041	0,001	0,059	0,002
Proteobacteria	Alphaproteobacteria	Rhizobiales	Unclassified	Unclassified	0,000	0,005	0,000	0,075	0,005	0,000	0,000	0,000	0,000	0,000	0,020	0,001	0,037	0,002
Proteobacteria	Alphaproteobacteria	Rhodobacterales	Rhodobacteraceae	Unclassified	0,000	0,000	0,005	0,021	0,000	0,005	0,000	0,000	0,000	0,000	0,007	0,001	0,010	0,002
Proteobacteria	Betaproteobacteria	Rhodocyclales	Rhodocyclaceae	Unclassified	0,000	0,000	0,000	0,000	0,000	0,000	0,000	0,000	0,000	0,005	0,000	0,001	0,000	0,002
Proteobacteria	Alphaproteobacteria	Rhodospirillales	Unclassified	Unclassified	0,000	0,059	0,000	0,011	0,000	0,000	0,000	0,000	0,000	0,000	0,017	0,000	0,028	0,000
Proteobacteria	Alphaproteobacteria	Rhodospirillales	Rhodospirillaceae	Unclassified	0,000	0,000	0,000	0,064	0,000	0,000	0,000	0,000	0,000	0,000	0,016	0,000	0,032	0,000
Proteobacteria	Gammaproteobacteria	Alteromonadales	Alteromonadaceae	Unclassified	0,000	0,000	0,016	0,000	0,000	0,000	0,000	0,000	0,000	0,000	0,004	0,000	0,008	0,000
Proteobacteria	Gammaproteobacteria	Thiotrichales	Unclassified	Unclassified	0,000	0,011	0,000	0,000	0,000	0,000	0,000	0,000	0,000	0,000	0,003	0,000	0,005	0,000
Proteobacteria	Alphaproteobacteria	Sphingomonadales	Sphingomonadaceae	Unclassified	0,000	0,000	0,005	0,000	0,000	0,000	0,000	0,000	0,000	0,000	0,001	0,000	0,003	0,000
Proteobacteria	Gammaproteobacteria	Vibrionales	Vibrionaceae	Vibrio	3,320	5,297	3,090	5,698	0,486	0,278	0,909	0,144	2,170	0,412	4,351	0,733	1,337	0,750
Proteobacteria	Gammaproteobacteria	Xanthomonadales	Xanthomonadaceae	Xanthomonas	0,000	0,016	0,000	0,000	0,000	0,000	0,000	0,005	0,000	0,000	0,004	0,001	0,008	0,002
Spirochaetes	Spirochaetia	Spirochaetales	Spirochaetaceae	Spirochaeta	0,251	0,743	0,401	0,128	0,102	0,198	0,134	0,053	0,476	0,641	0,381	0,267	0,266	0,236
Spirochaetes	Spirochaetia	Spirochaetales	Spirochaetaceae	Unclassified	0,337	0,102	0,374	0,000	0,021	0,096	1,016	0,075	0,171	0,037	0,203	0,236	0,181	0,386
Spirochaetes	Spirochaetia	Spirochaetales	Unclassified	Unclassified	0,000	0,000	0,016	0,000	0,000	0,000	0,000	0,000	0,000	0,000	0,004	0,000	0,008	0,000
SR1	SR1_genera_incertae_sedis	SR1_genera_incertae_sedis	SR1_genera_incertae_sedis	SR1_genera_incertae_sedis	0,011	0,091	0,069	0,000	0,016	0,000	0,000	0,000	0,000	0,000	0,043	0,003	0,044	0,007
Synergistetes	Synergistia	Synergistales	Synergistaceae	Cloacibacillus	0,000	0,000	0,000	0,016	0,000	0,000	0,000	0,000	0,000	0,000	0,004	0,000	0,008	0,000
Unclassified	Unclassified	Unclassified	Unclassified	Unclassified	1,342	1,219	0,754	0,545	6,634	4,378	2,267	0,663	1,091	4,731	0,965	3,294	0,377	2,334

Unclassified	Unclassified	Unclassified	Unclassified	Unclassified	0,000	0,032	0,000	0,000	0,000	0,000	0,000	0,000	0,011	0,000	0,008	0,002	0,016	0,004
Verrucomicrobia	Verrucomicrobiae	Verrucomicrobiales	Verrucomicrobiaceae	Akkermansia	0,005	0,000	0,000	0,000	0,000	0,000	0,000	0,053	0,000	0,000	0,001	0,009	0,003	0,022
Verrucomicrobia	Verrucomicrobiae	Verrucomicrobiales	Verrucomicrobiaceae	Luteolibacter	0,000	0,000	0,000	0,016	0,000	0,000	0,000	0,000	0,000	0,000	0,004	0,000	0,008	0,000
Verrucomicrobia	Opitutae	Opitutales	Opitutaceae	Opitutus	0,005	0,027	0,000	0,000	0,000	0,000	0,005	0,000	0,000	0,000	0,008	0,001	0,013	0,002
Verrucomicrobia	Spartobacteria	Spartobacteria_genera_incertae_sedis	Spartobacteria_genera_incertae_sedis	Spartobacteria_genera_incertae_sedis	0,000	0,000	0,021	0,080	0,000	0,005	0,000	0,310	0,000	0,011	0,025	0,054	0,038	0,125
Verrucomicrobia	Subdivision3	Subdivision3_genera_incertae_sedis	Subdivision3_genera_incertae_sedis	Subdivision3_genera_incertae_sedis	0,000	0,075	0,000	0,021	0,000	0,000	0,000	0,107	0,000	0,005	0,024	0,019	0,035	0,043
Verrucomicrobia	Subdivision5	Subdivision5_genera_incertae_sedis	Subdivision5_genera_incertae_sedis	Subdivision5_genera_incertae_sedis	0,000	0,123	0,037	0,000	0,000	0,000	0,000	0,000	0,000	0,000	0,040	0,000	0,058	0,000
Verrucomicrobia	Verrucomicrobiae	Verrucomicrobiales	Verrucomicrobiaceae	Unclassified	0,011	0,000	0,000	0,107	0,000	0,000	0,000	0,000	0,000	0,000	0,029	0,000	0,052	0,000
